# Ab initio framework for deciphering trade-off relationships in multi-component alloys

**DOI:** 10.1038/s41524-024-01342-2

**Published:** 2024-07-16

**Authors:** Franco Moitzi, Lorenz Romaner, Andrei V. Ruban, Max Hodapp, Oleg E. Peil

**Affiliations:** 1 Materials Center Leoben Forschung GmbH, Roseggerstraße 12, Leoben, A-8700 Austria; 2 Chair of Physical Metallurgy and Metallic Materials, Department of Materials Science, University of Leoben, Roseggerstraße 12, Leoben, A-8700 Austria; 3 Department of Materials Science and Engineering, Royal Institute of Technology, 10044 Stockholm, Sweden

**Keywords:** Computational methods, Electronic properties and materials

## Abstract

While first-principles methods have been successfully applied to characterize individual properties of multi-principal element alloys (MPEA), their use in searching for optimal trade-offs between competing properties is hampered by high computational demands. In this work, we present a framework to explore Pareto-optimal compositions by integrating advanced ab initio-based techniques into a Bayesian multi-objective optimization workflow, complemented by a simple analytical model providing straightforward analysis of trends. We benchmark the framework by applying it to solid solution strengthening and ductility of refractory MPEAs, with the parameters of the strengthening and ductility models being efficiently computed using a combination of the coherent-potential approximation method, accounting for finite-temperature effects, and actively-learned moment-tensor potentials parameterized with ab initio data. Properties obtained from ab initio calculations are subsequently used to extend predictions of all relevant material properties to a large class of refractory alloys with the help of the analytical model validated by the data and relying on a few element-specific parameters and universal functions that describe bonding between elements. Our findings offer crucial insights into the traditional strength-vs-ductility dilemma of refractory MPEAs. The proposed framework is versatile and can be extended to other materials and properties of interest, enabling a predictive and tractable high-throughput screening of Pareto-optimal MPEAs over the entire composition space.

## Introduction

Refractory multi-principal element alloys (MPEAs) have gained significant interest for high-temperature applications due to their excellent mechanical properties. These alloys consist of several high-melting-point elements such as tungsten (W), tantalum (Ta), molybdenum (Mo), zirconium (Zr), hafnium (Hf), and vanadium (V), that form body-centered cubic (bcc) solid solutions. Despite their high yield strength at ambient temperatures and remarkable strength retention at high temperatures above 1000 K^1–5^, refractory MPEAs are limited by their low tensile ductility and exhibit a sharp ductile-to-brittle transition^6–10^, which restricts their use to high-temperature applications, such as gas turbine engines^5,11^.

However, certain alloy compositions have been identified to have a more favorable combination of strength and ductility than others^12^. For instance, the ductilisation effect of rhenium (Re) and iridium (Ir) in tungsten is a well-known example that keeps the overall strength of the material virtually unchanged^13–15^. Consequently, the quest for discovering new compositions of refractory MPEAs with improved mechanical properties remains an important challenge in materials science. The conventional trial-and-error method of materials design is too laborious for complex MPEAs. This is where computer-guided design opens new opportunities for efficient exploration of the vast composition space of these materials.

In recent years, many works have introduced computational methods to identify and characterize alloys with tailored properties^16–23^, such as the solid solution strengthening, hardness, ductility index, and thermodynamic stability. Moreover, some of these methods have been employed in the context of the optimization of several target properties^16,24–26^, with the relevant material properties being obtained from various modeling approaches using experimental and theoretical input. For example, methods based on machine learning (ML) use various sets of descriptors partly derived from ab initio calculations in order to fit experimental data^27–29^. This approach, however, often requires large data sets and, most crucially, it is intrinsically interpolative and cannot give predictions outside the domain covered by an existing data set. A more transferable methodology is to use properties of elements, or simple compounds, for extrapolation of target properties to multi-component properties, mainly using the rule-of-mixture^30–32^. Despite its appeal due to its simplicity, this approach cannot capture the full complexity of a system in the case of intricate interactions (e.g., due to abrupt changes in the near-Fermi level band structure^33^).

A typical method of choice for truly predictive calculations of alloy properties is density functional theory (DFT). However, explicit evaluation of fracture or yield strength in alloys is mostly out of reach for available DFT implementations. Although this may become possible with general-purpose interatomic potentials^34,35^, a fairly robust description can be obtained by using appropriate models, which have been shown to accurately predict strengthening^36–38^ and ductility^39–42^. These models combine linear elasticity and microscopic principles, and the only required input parameters are certain material properties. Specifically, apart from basic equilibrium properties, such as the molar volume and elastic constants, the strengthening model requires misfit volumes related to concentration derivatives of the alloy volume, while the ductility model relies on surface and unstable stacking fault (USF) energies computed for specific orientations. Importantly, all of these parameters are accessible through simulations using first-principles methods, yet, such simulations are still challenging for disordered MPEAs.

The most common approach to ab initio alloy modeling is to represent a disordered alloy using special quasi-random structures (SQS) or similar supercell representations that try to mimic perfect randomness^43–45^. However, this method has several inherent limitations for MPEAs. First, the computational effort grows exponentially as the number of alloy components increases. Moreover, properties influenced by global symmetry or local chemical environment^46,47^, such as surface energies or elastic constants, present significant challenges for convergence and necessitate the use of even larger cells, limiting the number of components in the best case to three or four. Calculating properties for arbitrary compositions, especially when components are present in low concentrations, is even more cumbersome.

One alternative methodology is the coherent potential approximation (CPA)^48,49^, whose advantages are its extremely small computational expenses, irrespective of the number of components, as well as its ability to treat arbitrary alloy concentrations, providing a straightforward way for accurate evaluation of concentration derivatives. Unfortunately, the single-site nature of CPA renders it impossible to take into account effects of local atomic relaxations that are crucial for a correct characterization of defects, such as USFs, or inhomogeneous structures, e.g., surfaces.

Another alternative to traditional supercell methods are machine-learning interatomic potentials (MLIPs) that are trained on ab initio data^50–53^. The size of supercells is not limited with MLIPs, which is especially important when simulating extended defects like interfaces, where proper configurational averaging becomes an issue with MPEAs. In contrast to the previously proposed way of passively training general-purpose interatomic potentials on a pre-defined fixed training set (e.g.^34,35,54,55^), whose accuracy is not sufficient for our goals, especially for parts of the configurational space not covered by the training set, the use of active learning enables automatic generation of MLIP training sets specifically tailored to a given class of systems and a given set of properties. This allows one to predict any material property of interest with DFT accuracy over the entire composition space of an alloy^39,56^ with a minimal amount of DFT calculations.

In this work, we combine the two approaches—CPA and actively learned Moment Tensor Potentials (MTP), a class of MLIPs—to characterize solid solution strengthening and ductility of refractory MPEAs. In particular, we use CPA to compute the elastic constants and misfit volumes, needed for computing the critical resolved shear stress (CRSS) within the model of Maresca and Curtin^36,37^. MTPs are used to simulate large supercells, necessary to calculate surface and USF energies which are needed to parameterize a model for intrinsic ductility based on the Rice-Thomson theory^40^.

The crucial point of our approach is to enhance the simulation efficiency by employing the most suitable method for different sets of properties. Specifically, CPA’s quantum mechanical treatment of solid solutions and its analytical nature eliminates finite-size cell effects and sampling errors related to random atomic configurations, making it excel in calculations of the concentration dependence of properties that are relatively insensitive to local relaxations, such as elastic constants and misfit volumes. In comparison to supercell calculations with MLIPs, CPA is especially superior in accuracy when dealing with abrupt changes of the Fermi-surface topology, which is often encountered in bcc alloys and can have a significant impact on the concentration dependence of properties. On the other hand, ab initio based MTP methodology surpasses CPA in dealing with defects and special geometries, such as surfaces, due to its capability to incorporate local atomic relaxations.

Another important part of our framework is a simple model that is only slightly more complicated than the rule of mixtures but can accurately capture the concentration dependence of all the quantities relevant for strengthening and ductility over the entire composition space of a large class of refractory alloys. The model is based on simple element-specific descriptors and universal functions of the *d*-electron valence, with a few adjustable parameters that can be easily fitted to data from simple compounds. Importantly, the model is based on fundamental properties of bonding in transition metals, which makes it capable of extrapolative predictions, rendering it as a potentially useful tool for exploring the *entire* space of refractory MPEAs. Moreover, the analysis of higher-order contributions beyond the first order can provide an insight into interactions between individual alloy components.

As a proof of concept for the flexibility of such an approach, we perform multi-objective optimization (MOO) of strength and ductility for three- and four-component alloys. Then, we use these results to validate the model and extend our predictions to a larger class of alloys. By analyzing the results, we reveal several potential pitfalls of conventional methods and show how they are easily overcome within our approach.

## Results

### Multi-objective optimization of strength and ductility

We consider homogeneous alloys whose strength can be characterized by the CRSS due to impeded mobility of dislocations interacting with the alloy components that act as a random field of solutes. Within the Maresca-Curtin model^37^, the CRSS is given by *τ*_*y*_ ≡ *τ*_*y*_(*V*, {Δ*V*_*n*_}, *C*_*ij*_), where *V* is the specific volume, Δ*V*_*n*_ are the misfit volumes for each component *n*, and *C*_*ij*_ are the elastic constants (see Methods 4.2 for details). If the material parameters are obtained from first principles, the model allows for parameter-free predictions of yield strength without relying on experimental input.

The strength of a solid solution is known to compete with its ductility. The ductility of a refractory MPEA is mainly determined by the intrinsic fracture behavior of single-crystalline grains. The ability of a material to undergo plastic deformation affects the way unavoidable defects grow to cracks and propagate through the material. If a crack is blunting (propagating) upon external loading, the material is said to be intrinsically brittle. If, instead, a dislocation is emitted from the crack tip, the material is said to be intrinsically ductile. This competition between blunting and dislocation emission on active slip systems in bcc alloys can be quantified using the Rice-Thomson model^40,57^. Within the Rice-Thomson model, ductility is described by means of a ductility index, *D*, which depends on the ratio of the unstable stacking fault energy *γ*_USF_ and the surface energy *γ*_surf_, times a prefactor depending on the crack orientation and the elastic constants. In this theory, lower *D* values indicate increased alloy ductility, and *D* < 1 signifies the absence of intrinsic brittleness. A recent application of the ductility index by Mak et al.^41^ has led to the development of an RT ductility criterion for refractory metal alloys. Their study showcased the correlation of *D* with measured fracture strain and its predictive capability for ductility trends.

**Fig. 1:**
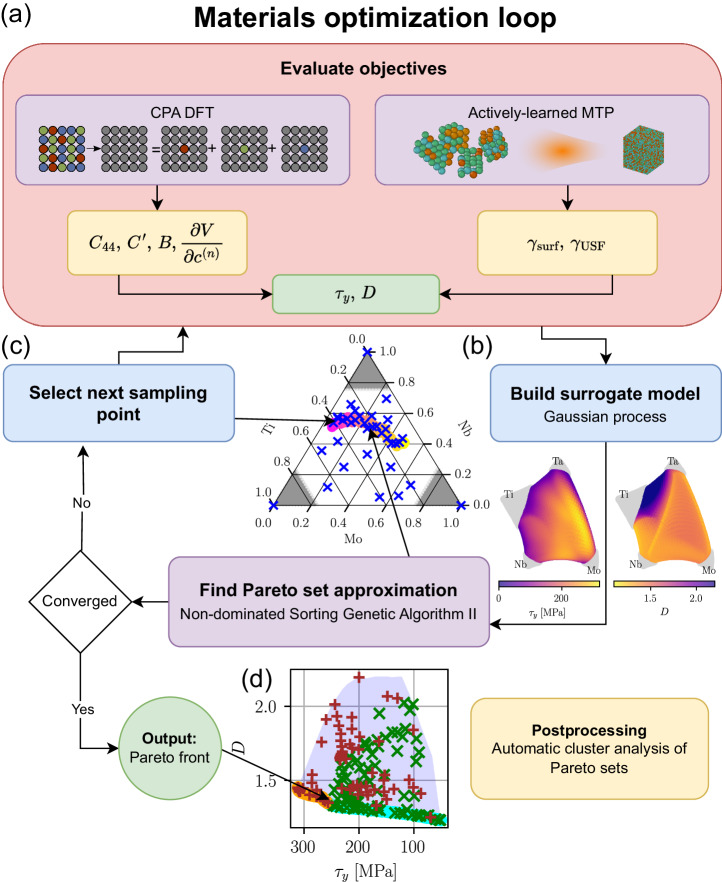
Flowchart of the materials design loop using Bayesian multi-objective optimization. **a** Advanced ab initio techniques such as the coherent potential approximation (CPA) and actively-learned moment tensor potentials (MTPs) to calculate the strength, *τ*_*y*_, and ductility, *D*, objectives. Quantities obtained from ab initio are: Shear, *C*_44_, ${C}^{{\prime} }$, and bulk, *B*, moduli, specific volume (per atom), *V*, and its concentration derivatives, ∂*V*/∂*c*^(*n*)^, all evaluated at room temperature; the surface energy, *γ*_surf_, and the USF energy, *γ*_USF_. **b** Employing Gaussian process regression, we construct a surrogate model, and a genetic algorithm is applied to the search of Pareto-optimal compositions. **c** The sampling points are chosen from the Pareto front approximation until convergence is reached. **d** Pareto-optimal alloys are the key outcome of the process.

**Fig. 2:**
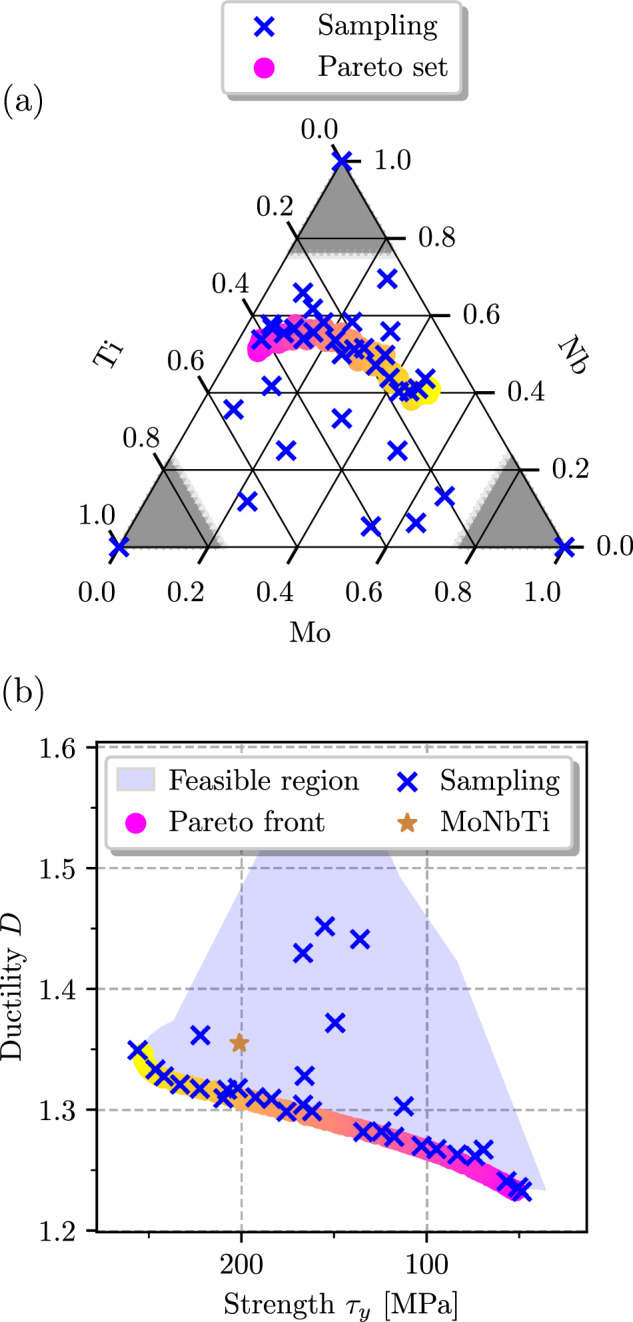
Optimization results for the MoNbTi system. **a** Pareto set approximation (PSA) alongside with sampling points in the search space. **b** Pareto front approximation (PFA), sampling points and feasible region in objective space. PFA was gradually color-coded from yellow, which corresponds to high strength, to pink, which corresponds to high ductility. PSA uses the same color coding.

Improved ductility is expected for alloys with lower stacking fault energies, which facilitates dislocation emission. At the same time, the opposite is true for improved strength, and to investigate this competition, we leverage a Bayesian multi-objective optimization framework to uncover sets of Pareto-optimal compositions, with the objective functions obtained from first principles. The flowchart in Fig. 1 presents an overview of the workflow developed in this work. The two main ingredients of the workflow are (1) the calculations of the objectives, the CRSS, *τ*_*y*_, and the ductility index, *D* (Fig. 1a), and (2) a Bayesian optimization loop based on the non-dominated sorting genetic algorithm (NSGA-II)^58^ for finding the Pareto-set approximation (Fig. 1b, c); see Methods 4.4 for details. The key feature here is that NSGA-II utilizes an intermediate surrogate model (Gaussian process in our case), significantly reducing the need for direct evaluations of the objective functions.

As shown in Fig. 1a, the quantities obtained from first-principles calculations are the elastic constants, *C*_44_, ${C}^{{\prime} }$, and *B*, the concentration derivatives of the specific volume, and the planar defect energies: the surface energies, *γ*_surf_ ({100}, {110}), and the unstable stacking fault energy, *γ*_USF_ ({110}, {112}), evaluated for relevant orientations {•}^41^.

We use CPA to calculate the elastic constants and volumes because these quantities are insensitive to local atomic relaxations, and evaluation of concentration derivatives is much more efficient with this approach compared to SQS calculations. Furthermore, a similar methodology has already been successfully applied to computing CRSS of iron-group MPEAs^59,60^.

On the other hand, the planar defects are strongly affected by local lattice relaxations, and we, therefore, resort to a supercell approach for these quantities. However, carrying out high-throughput calculations with the conventional SQS is computationally unwieldy, especially for properties requiring non-trivial structure relaxations (e.g., surfaces). Therefore, we use actively-learned MTPs that are able to predict material properties with near-DFT accuracy^39,56^, requiring only a small fraction of the computational expenses of a direct DFT calculation. This allows us to perform structure relaxation on supercells with hundreds of thousands of atoms at a cost that is only slightly higher than that of classical interatomic potentials, such as the embedded atom method. In comparison to previously published works^27,41,61,62^, our approach consistently produces comparable USF energies for specific alloy compositions (see Results and Discussion) but we can also treat any other composition of the alloy once the MLIPs are trained. Moreover, we anticipate greater accuracy for systems affected by Fermi surface nesting^63^ or for alloys experiencing local lattice reconstructions^64^ because our methodology does not impose constraints on cell size or lattice relaxations. Indeed, in case of surface energies, the differences between MTP-based and SQS calculations become much more pronounced (sometimes, > 200 mJ/m^2^, which is > 10% of the typical value of *γ*_surf_), mainly due to a considerably slower convergence of the surface energy with the cell size, as will be discussed in more details later on (see Section 3).

### Search for Pareto-optimal alloys

We now combine the calculations of the CRSS and the ductility index, *D*, and perform a search for Pareto-optimal alloys in the composition space, according to the workflow shown in Fig. 1. We first analyze the MoNbTi alloy, whose search (design) space can be easily visualized using ternary diagrams. We constrain the search space to a concentration range of 0.0 ≤ *c*_*i*_ ≤ 0.75 for each component *i*. This is motivated by the fact that pure Ti is stable in the hcp structure (*α*-Ti) under ambient conditions and it has limited miscibility with the other two elements, Mo^65^ and Nb^66^ (for more detailed analysis of phase stability, see Supplementary Information). Moreover, dilute alloys offer minimal potential for solid solution strengthening.

The constraints are handled by penalty functions within NSGA-II, which penalize undesirable solutions by reducing their fitness values in proportion to the degree of constraint violation. In principle, this approach could be extended to define more complex regions in the search space that should be avoided, e.g., regions of thermodynamic instability or other unfavorable properties.

Figure 2a shows the sampling points in the search space of MoNbTi, alongside with the Pareto set approximation (PSA), which is a set of compositions that are non-dominated by each other but are superior to the other compositions in the Pareto sense. This can be seen more clearly in Fig. 2b, where we show the Pareto front approximation (PFA) at the bottom of the feasible region, resulting from the sampling in the objective space. There is a continuous mapping between PFA and PSA. The PFA’s left portion corresponds to PSA’s right portion, and vice versa. On the right side of PSA and the left side of PFA are stronger but less ductile alloys. In both PFA and PSA, the color-coded scheme designates yellow for high strength and pink for high ductility. Generally, we observe that relative changes in the ductility index across the concentration space are not as severe as changes in the strength. The lower limit of the strength for Pareto-optimal alloys within the constraints given above is about 50 MPa, while the strongest alloys have *τ*_*y*_ ≈ 260 MPa. On the other hand, the corresponding ductility index, *D*, changes from 1.23 – 1.35 (with the error of *D* estimated to be < 0.015, see Supplementary Information). It is also worth noting that the Pareto set avoids the region around the equimolar composition.

Regions of higher strength can be found close to the equimolar MoNb alloy with small additions of Ti. Reducing the Mo content in the alloy enhances its ductility and reduces the likelihood of brittle fracture. On the other hand, the alloy must contain a certain amount of Mo to ensure a reasonable strengthening effect. More ductile alloys can be found in a region close to the Nb_0.6_Ti_0.4_ binary. The improved ductility comes at a price of the strength that is almost five times smaller than the maximum value.

**Fig. 3:**
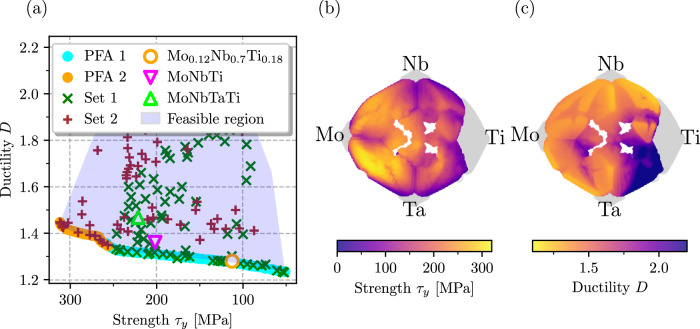
Optimization results for the MoNbTiTa system. **a** Visualization of the PFA and sampling points. The Pareto set comprises two distinct regions in the concentration space derived from automated clustering. These regions define the corresponding sections (PFA1) and (PFA2) on the Pareto front. The sampling points are color- and marker-coded based on their proximity to the respective PFA regions. Open symbols mark specific alloys mechanically tested in refs. ^68,69^. *t*-SNE projections of (**b**) ductility index *D* and (**c**) strengthening *τ*_*y*_ for the whole search space. Gray areas are beyond the search space. Sets 1 and 2 mark sampling points that are closer in the search space to the points of PFA1 and PFA2, respectively.

Pareto-optimal alloy compositions in the three-component MoNbTi alloy system exhibit a continuous and smooth transition across the entire design space, forming a connected Pareto set. To investigate non-trivial alloying effects, we expand the composition space by introducing Ta, anticipating Fermi surface nesting effects caused by Ta alloying. Similarly to the case of MoNbTi, we confine the search space to concentration values ranging from 0.0 – 0.75 for each component, with the exception of Ti, which is restricted to a maximum atomic fraction of 0.55. This limitation is motivated by the limited solubility of Ti, as described in ref. ^67^ (see also Supplementary Information). The results of the optimization, represented by the PFA and sampling points, as illustrated in Fig. 3a reveal a more intricate concentration dependence of the objectives, which can also be seen in the *t*-SNE projections displayed in Fig. 3b, c (see Methods 4.5 for details).

These plots indicate that, while the ductility index, *D*, is, again, minimal near binary NbTi, the strength, *τ*_*y*_, is maximal in two regions: (1) close to binary MoNb, with small additions of Ti, as in the MoNbTi case; (2) the vicinity of binary MoTa, which exhibits considerably higher strength than the 3-component alloy. This leads to two effectively disjoint Pareto subsets in the composition space of the MoNbTiTa alloy.

Indeed, clustering analysis has detected that parts of the Pareto front denoted by PFA1 and PFA2 in Fig. 3a can be associated with two separate regions in the concentration space. Despite a seemingly continuous crossover between these two regions in the objective space, the regions are truly disjoint in the composition space. The right, ductility-favoring portion of the Pareto front, PFA1, corresponds to alloys similar to the three-component MoNbTi system with minor additions of Ta. The optimal compositions in this region are characterized by the Nb content ranging from 0.4 – 0.6, by the Ti content ranging from 0.2 – 0.4, and moderate Mo concentration maintaining the strengthening. At the same time, the left portion of the Pareto front, PFA2, represents a separate region comprising alloys with high Mo (0.4 − 0.6) and moderate Ta (0.2 − 0.35) content, whereby Ta is responsible for a strength above 250 MPa, i.e., beyond those reachable by the MoNbTi system. An important feature of the PFA2 is a small knee corresponding to *τ*_*y*_ ≈ 270 MPa. Starting from this point, one can either improve ductility without reducing the strength much, or enhance the strength without compromising on ductility.

In Figs. 2a and 3a, we highlight three alloys from refs. ^68,69^ which were mechanically tested. Both the equimolar MoNbTiTa and MoNbTi exhibit similar strength in experimental measurements, with the four-component alloy showing slightly higher strength. The Nb-rich Mo_0.12_Nb_0.7_Ti_0.18_ alloy demonstrates ~ 2/3 of the strength of the others. This behavior is also observed in our results. Additionally, ductility was indirectly assessed in these experimental works by measuring strain at fracture. The Nb-rich alloy exhibited the highest ductility, while the other two alloys showed lower ductility, yet remained non-brittle. Our ductility index corroborates this finding. All three alloys are located near the Pareto-front, with the Nb-rich alloy being located precisely on the ductile portion of the Pareto front. For MoNbTiTa, our results suggest that increasing the Mo and Nb content, while adjusting the Ta content, can improve the ductility of the equimolar MoNbTiTa alloy, without sacrificing the strength.

### Virtual bond approximation model

So far, the main idea of the workflow was to get accurate and predictive results with high computational efficiency. Still, characterizing multiple alloy systems in this ways would require a lot of DFT calculations (MTP training and CPA). Instead, we would like to use the results obtained thus far for MoNbTiTa to analyze the strengthening and ductility behavior in a broader range of refractory alloys. One way to achieve this would be to define a set of “good” descriptors and train a black box ML model, which can then be used to obtain properties at arbitrary compositions. Such descriptors may encompass purely local structural characteristics^70–72^, effective interatomic interactions^73^ or local electronic structure-related properties like moments of the local density of states and energies of simpler substructures^27,74^. One significant disadvantage of such data-driven models is that they often lack interpretability and they are also only interpolative, meaning that one has to have a large amount of data covering enough of alloy systems to be able to predict properties of an alloy not included in the initial data set. Physical models, on the other hand, are more transferable because they require fewer data points for training and provide clear insights into system behavior, fostering a deeper understanding of cause-and-effect relationships. Moreover, such models based on fundamental principles can be also considered as extrapolative, which prevents implausible outcomes, ensuring reliability.

**Fig. 4:**
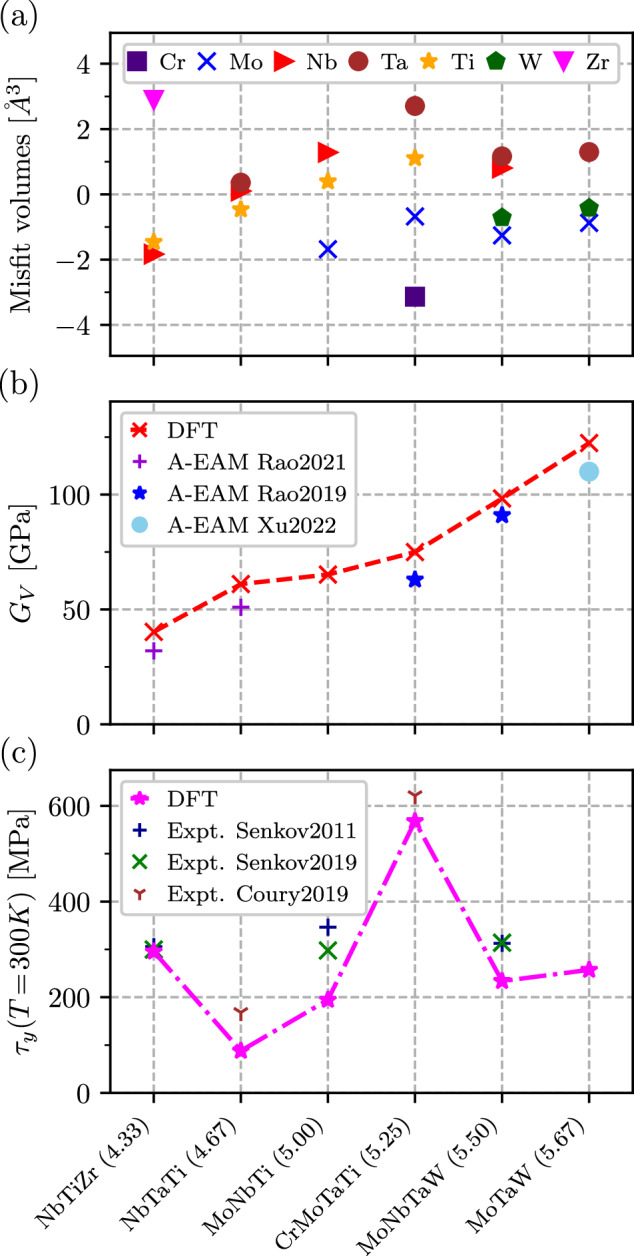
Strength-related properties for various equimolar alloys. Comparison of misfit volumes Δ*V*_*i*_ (**a**), the shear modulus, *G*_*V*_, (**b**), and the solid solution strengthening, *τ*_*y*_ (**c**) for various refractory alloys. The alloys are sorted according to their valence electron concentration (VEC). Experimental values for *τ*_*y*_ are from refs. ^68,119,120^. Shear modulus is compared to results obtained with *A*-atom embedded-atom potential from refs. ^121–123^.

One of the simplest examples of a physics-motivated model is a correlation between the valence electron count (VEC) and the ductility. In particular, a lower VEC is known to be associated with an enhanced intrinsic ductility due to a greater elastic softness^11,41,42,75^. Brittle alloys are prevalent at a VEC of around 5 – 6, with an improved ductility beyond this range^75^. A similar correlation can be seen for the shear modulus, *G*, as shown in Fig. 4b. The shear modulus increases more significantly than the bulk modulus, resulting in a larger shear-to-bulk modulus ratio (*G*/*B*) at higher values of VEC. This trend is frequently associated with embrittlement.

**Fig. 5:**
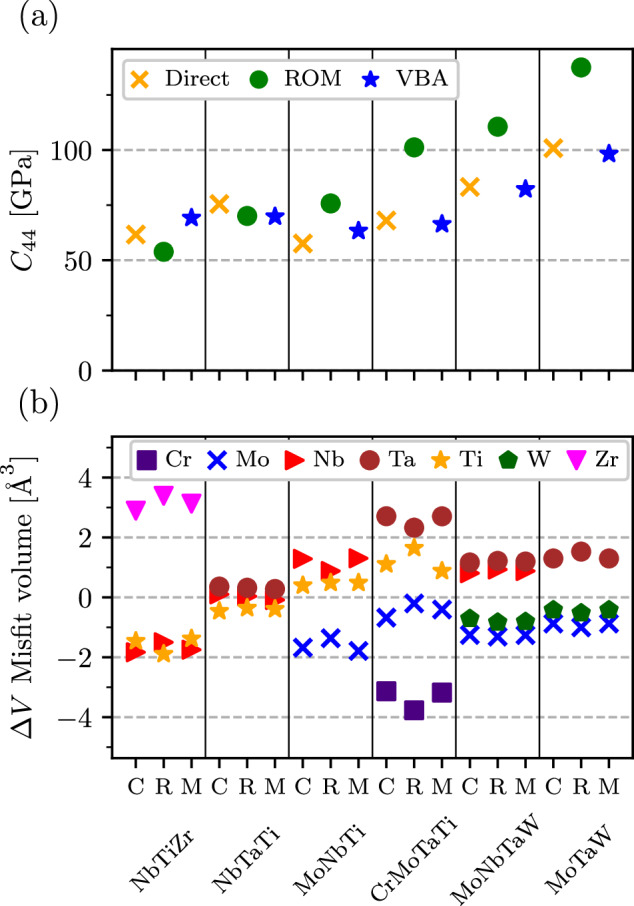
Elastic modulus C_44_ and misfit volumes for various equimolar alloys. Comparison of (**a**) *C*_44_ and (**b**) Δ*V* misfit volumes obtained from the rule-of-mixture (R), virtual bond approximation (M), and direct calculations (C).

Despite its clear physical meaning as the bonding state filling factor, the VEC does not take into account interactions between alloy components. This, in particular, can explain why it fails to reveal any particular trend in misfit volumes (Fig. 4a) and it is, thus, not a useful descriptor for the CRSS, as can be seen in Fig. 4c. Another popular model – the rule of mixtures (ROM), also known as Vegard’s law when applied to the lattice constant or volume – shares the same drawback (see, e.g., Fig. 5).

Our approach is based on a slightly modified version of the virtual bond approximation (VBA)^76^ that can be considered as a generalization of classical bonding theory of transition metals^77^ to alloys. The key idea of the VBA is to assign universal functions to local bonds between alloy components. These so-called *virtual bond* functions depend on the average *d*-valence of elements forming the bond, as well as element-specific bandwidths (equivalently, second moments of the density of states) of the corresponding elemental compounds. To second order in inter-component interactions (possible extension to higher orders is straightforward), the VBA takes the form

1 $$\begin{array}{l}{{{\boldsymbol{\xi }}}}(\{{c}_{i}\})\,=\,\sum\limits_{i}{c}_{i}\,{w}_{i}\,{{{{\boldsymbol{\xi }}}}}^{(1)}\left({N}_{i}\right)+\\\qquad\qquad+\,\sum\limits_{ij}{c}_{i}\,{c}_{j}\,{w}_{ij}\,{{{{\boldsymbol{\xi }}}}}^{(2)}\left({N}_{ij}\right),\end{array}$$

where **ξ**({*c*_*i*_}) stands for *B*, ${C}^{{\prime} }$, *C*_44_, *V*, or Δ*E* (for USF and surface energies), with *c*_*i*_ being atomic fractions of components and **ξ**^(*k*)^ representing the corresponding virtual bond functions of the average *d*-valences, *N*^(*k*)^ ≡ {*N*_*i*_, *N*_*ij*_}, and of bandwidth parameters *w*^(*k*)^ ≡ {*w*_*i*_, *w*_*ij*_} (see Methods 4.6 for details). Note that *N*_*i*_ and *w*_*i*_ are element-specific parameters, while the second-order parameters are defined as *N*_*ij*_ = (*N*_*i*_ + *N*_*j*_)/2 and ${w}_{ij}=\sqrt{{w}_{i}{w}_{j}}$.

The unknowns **ξ**^(*k*)^ are obtained using linear regression over a set of values of the associated quantity computed for a series of systems including pure elements (Cr, Mo, Nb, Ta, Ti, W, Zr) and equimolar binary alloys in the bcc structure. Since the inclusion of Ta causes deviations and abrupt changes in material properties attributed to Fermi surface transitions (e.g., see Supplementary Information), we also calculate several three- and four-component alloys containing Ta. Significant deviations from both directly calculated and ROM values, serve as a reliable indicator of whether additional alloys should be included. This procedure was only deemed necessary for elastic properties. The fitted model is applicable to any alloy composed of the seven mentioned elements.

A comparable approach was presented by Ferrari et al.^20,78^ where energies and material properties were fitted using polynomial functions of concentrations. A notable advantage of our method lies in the fact that we do not require parameters to be fit individually for each alloying system. Our approach promotes the coefficients from system-specific tabulated values to transferable parameters that encapsulate local bonding characteristics. For example, the VBA applied to iron-group MPEAs produced good results despite major complications due to the complex magnetic behavior of this class of alloys^59^.

To demonstrate the performance of our VBA model, we compare direct and VBA calculations for the most important properties for six previously considered equimolar alloys: NbTiZr, NbTaTi, MoNbTi, CrMoTaTi, MoNbTaW and MoTaW. The results are presented in Fig. 5, where we compare calculated (labeled as “C”), VBA model ("M”), alongside ROM ("R”) values for the misfit volumes, *C*_44_, and *γ*_surf_. The misfit volumes for equimolar compositions (Fig. 5b) are reproduced well by both the VBA model and the ROM, with the former giving systematically better values. However, the true advantage of using a model that includes inter-component interactions, becomes apparent when the composition is varied: In this case, the ROM often fails to reproduce the concentration trends, while the VBA model produces almost exact results corresponding to direct calculations (e.g., see Supplementary Information).

Obtaining virtual bond functions, *ξ*^(*k*)^, for surface and USF energies is more difficult due to relaxation effects. To take them into account, we apply an additional rescaling of the functions, as described in Methods 4.6. Test results can be found in Supplementary Information.

**Fig. 6:**
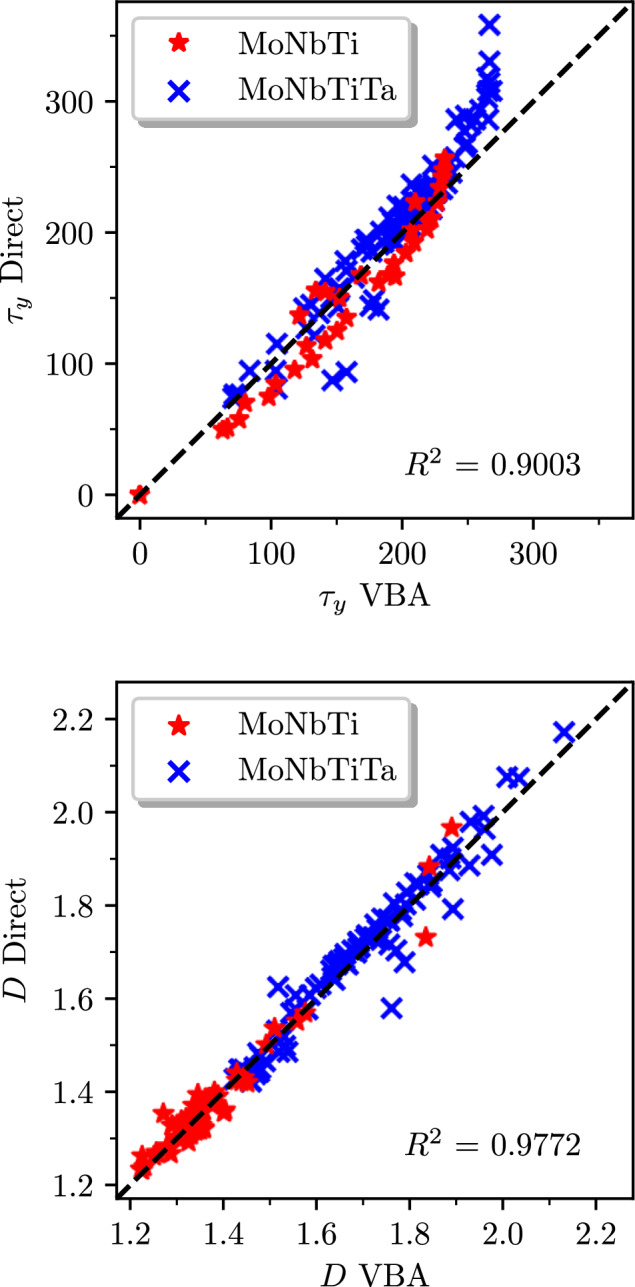
Parity plots showing strength and ductility calculated directly and from the virtual bond approximation for various alloys from the MoNbTi and MoNbTiTa system. The upper panel shows the correlation for strength, while the lower panel shows the correlation for ductility. D and τ_y_ refer to the directly calculated values, and *D*_VBA_ and *τ*_yVBA_ refer to the values from the virtual bond approximation.

To validate our VBA model against our ab initio results, we use the compositions previously generated during the optimization process for the MoNbTi and MoNbTiTa alloy systems, compute *τ*_*y*_ and *D* using the material parameters obtained from the VBA model, and compare the results with direct CPA+MTP calculations. As can be seen from Fig. 6, the agreement is remarkably good for such a simple model, with minor deviations due to its inability to replicate strong non-linear effects in the properties upon changing the concentration. A more detailed analysis reveals that most of the discrepancies (especially for *τ*_*y*_ in Fig. 6) are associated with Ta causing substantial non-linearity in the concentration dependence of the elastic properties (see Supplementary Information). Such a non-linear behavior is due to Fermi-surface effects and pose a challenge to any simple model.

The validation on MoNbTiTa has shown the ability of the VBA model to to describe trends in multicomponent alloys based on the data from elemental and simple alloy compounds. However, a much stricter test (also addressing an eventual overfitting issue) would be to check if the model can predict properties for alloys containing elements not included in the training set. With this in mind, Hf and V (or any alloy containing them) have been excluded from the training set used to fit parameters *ξ*^(*k*)^. To evaluate properties for alloys involving these elements, the only input needed is the corresponding bandwidth parameter (corrected for the row in the periodic table) and the number of *d*-electrons, which are element-specific constants.

**Fig. 7:**
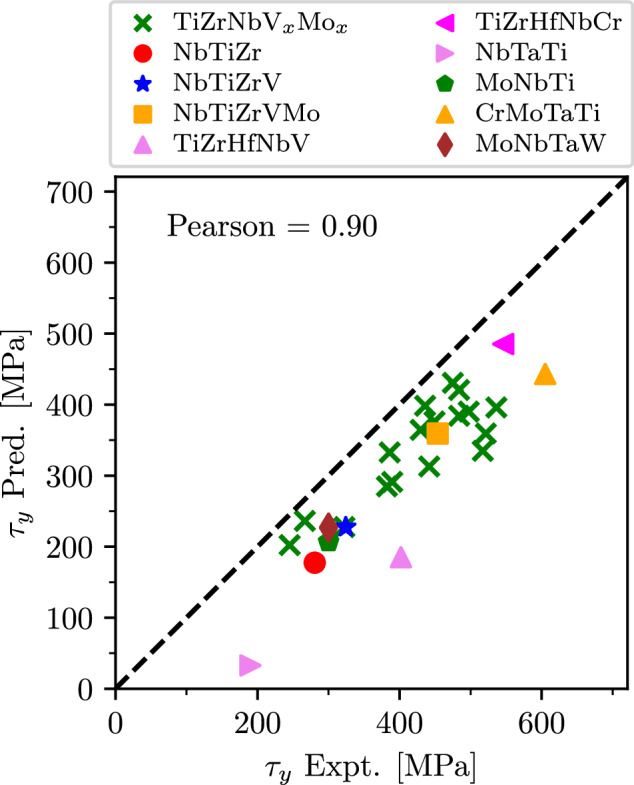
Comparsion between predicted and experimental strength for various alloys. Parity plot of predicted and experimentally obtained CRSS, *τ*_*y*_, for various alloys, including alloys containing Hf and V.

The model is then applied to a series of alloys containing nine *d* elements from three groups: Ti, V, and Cr. The results for the CRSS are shown in Fig. 7 where they are plotted against experimentally measured *τ*_*y*_^79–82^ (tabulated data can be found in Table S2 of Supplementary Information). One can see that the model captures trends very well in a large range of V-containing alloys. Some alloys display underestimated predictions, but the overall strength ranking remains accurate, reflected in a high Pearson coefficient. The overall underestimation is anticipated as the strengthening model gives the lower bound, as discussed in more details in Supplementary Information (the effect can also be seen in Fig. 4).

**Fig. 8:**
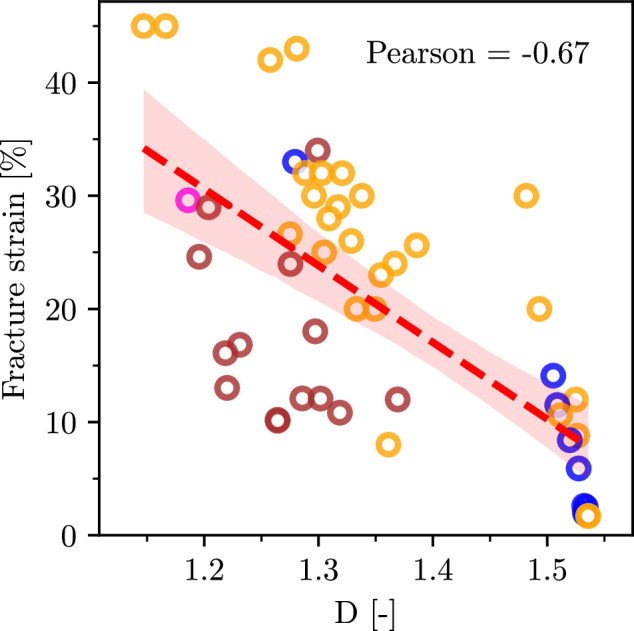
Correlation plot of predicted *D* and fracture strain for various alloys from ref. ^23^. Alloys containing Hf or V are marked with orange and brown dots, respectively. Alloys containing both Hf and V are marked with pink dots. The red area indicates the 95% confidence interval for the correlation between fracture strain and D.

The predicted ductility index is much more difficult to validate, as it is not a measurable quantity. Therefore, we compare it against the fracture strain for a range of alloys from Singh et al.^23^. As one can see from the results in Fig. 8 (tabulated data can be found in Supplementary Table 1), there is a significant anti-correlation between the measured strain at fracture and *D*, with the Pearson coefficient of -0.67. In critically assessing this outcome, one should keep in mind that it captures not only errors of the VBA model itself but also the qualitative nature of the ductility index, which is not directly related to the fracture strain, but rather, it is an indicator for the tendency to embrittlement. Furthermore, the *D*-criterion does not apply to intergranular fracture that can be facilitated by elements segregating at grain boundaries and reducing their cohesive strength. Nevertheless, it is important that most of the alloys on the brittle and ductile ends (having extreme values of *D*/fracture strain) are ranked correctly. Note also, that our results are very similar to an analogous plot of the fracture strain versus the ductility index in Singh et al.^23^, if one takes into account that the ductility index in the reference is related to the inverse of our *D*. Overall, the prediction results suggest that the VBA is sufficiently accurate to be used for a qualitative assessment and analysis of ductility-strength trade-offs for refractory MPEAs, as will be discussed in the next section in more details.

## Discussion

Although our results on the CRSS and the ductility index are qualitatively consistent with previously published calculations, there are quantitative differences, which we now analyze by examining individual quantities involved in the models.

The key parameters for calculating the ductility index, *D*, are the energies of USF (for two orientations, {110} and {112}) and the surface ({100} and {110}). In this context, we compare the results of equimolar MoNbTi of our calculations with actively-learned MTPs to respective values from previous studies in Table 1.

**Table 1:** Comparison of stacking fault and surface energies (in mJ/m^2^) for MoNbTi obtained from actively-learned MTP (this work), MTP trained on large data set^61^, SQS^41,62^ and surrogate model trained on SQS data^27^

	${\gamma }_{surf}^{\{100\}}$	${\gamma }_{surf}^{\{110\}}$	${\gamma }_{USF}^{\{110\}}$	${\gamma }_{USF}^{\{112\}}$
This work	1989	1832	730	853
Mak^41^	2317	2171	820	927
Zheng^61^	–	–	774	–
Xu^62^	–	–	765	865
Hu^27^	–	2189	817	–

In particular, the USF are often used in theories predicting the mechanical properties of bcc metallic alloys, and its energy is therefore often studied. Our MTP approach yields values of 730 *mJ*/*m*^2^ and 853 *mJ*/*m*^2^, for {110} and {112} respectively. The values for both orientations converge quickly with cell size, which validates the use of smaller cells in SQS calculations.

The SQS-based references values of Xu et al.^62^ and values from MTPs trained on the large data set of Zheng et al.^61^ render similar values, with rather small difference up to 40 *mJ*/*m*^2^. Errors on this scale can be attributed to the choice of a specific lattice parameter, statistical sampling errors and method-specific numerical errors (Supplementary Information contain a detailed analysis of errors for surface and USF energies). Only Mak et al.^41^ shows consistently higher values for all orientations. Notably, our analysis reveals the origin of the discrepancies among the reference values reported in the literature. Xu et al.^62^ and Zheng et al.^61^ calculated USFs by keeping the cell cubic with the lattice parameter of the alloy. In contrast, Mak et al.^41^ relaxed the out-of-plane lattice vector to zero stress, along with the ionic positions perpendicular to the stacking fault, while keeping the fault plane lattice vector fixed. Replicating this procedure with our MTP on similar cell sizes revealed a systematic increase of ~70–100 *mJ*/*m*^2^ in *γ*_USF_ for various compositions. In comparison, alternative approaches, such as the surrogate model of Hu et al.^27^, give comparable, but overestimated, values (the last row in Table 1). Additionally, our calculations highlight the substantial contribution of local ionic relaxations to the USF energy, causing an energy change of about 25%. Despite this, neglecting local relaxations does not affect overall trends and relative changes upon concentration variations, potentially justifying the use of CPA calculations for qualitative studies (more detailed analysis provided in Supplementary Information).

In contrast to USF energies, surface energies for random alloys are much more challenging to compute, hence there are relatively few references available. One of the reasons is the presence of surface reconstructions, which can result in contracted or extended bond lengths near surfaces. Lattice spacing only gradually recovers to the bulk spacing, making it necessary to use relatively large cells for accurate calculations. In addition, finite size ordering effects on the surface must be avoided for random alloys.

**Fig. 9:**
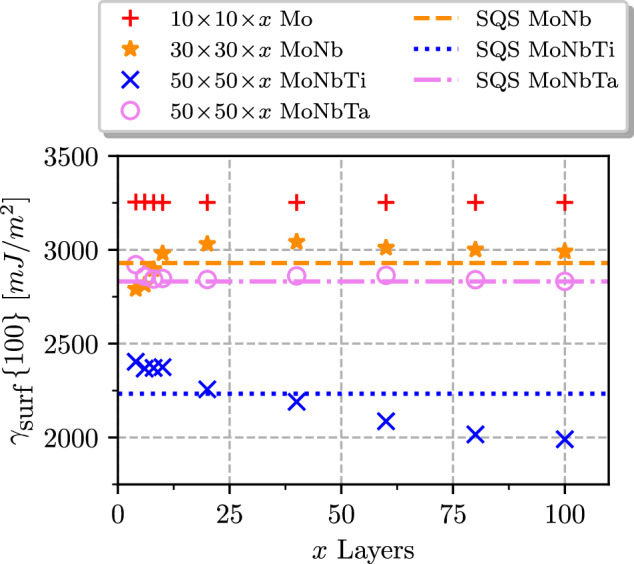
Cell size convergence. Convergence of surface energies with respect to the number of layers for pure Mo, equimolar MoNb, MoNbTi, and MoNbTa, along with corresponding results for an SQS cell sized 4 × 4 x 10. All values are obtained with MTPs.

According to Mak et al.^41^, the {100} and {110} surfaces of MoNbTi exhibit surface energies of 2317 *mJ*/*m*^2^ and 2171 *mJ*/*m*^2^, respectively. Moreover, the surrogate model of et al.^27^ has determined the surface energy for the {110} surface to be 2189 *mJ*/*m*^2^. Both results were obtained with the SQS approach. However, our methodology has produced lower values for these surface energies. Specifically, our calculations yield surface energies of 1989 *mJ*/*m*^2^ and 1832 *mJ*/*m*^2^ for the {100} and {110} surfaces, respectively. To understand the source of such a considerable discrepancy, we performed the convergence analysis with respect to the slab size (number of layers), which can be easily done with MTP. The analysis, whose results are presented in Fig. 9, clearly shows that the convergence of the surface energies can be rather slow. In the case of MoNbTi, up to 100 layers are required to produce the correct (converged) values. A more detailed examination of this behavior suggests that it can be traced back to a strong effect of statistical fluctuations of local atomic configurations on the relaxation contribution to the total energy (see Supplementary Materials for details). This emphasizes the importance of the analysis of supercell size convergence and also shows insufficiency of typical SQS sizes for accurate surface energies of random alloys.

**Fig. 10:**
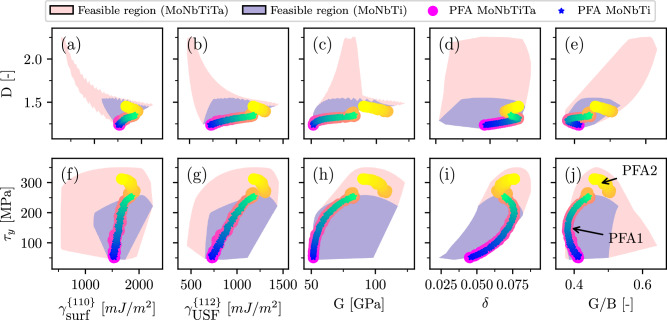
Feasible regions and correlations between materials parameters for MoNbTi and MoNbTiTa. The feasible regions (light blue for MoNbTi, light red for MoNbTiTa) and correlations between materials parameters (surface energy, ${\gamma }_{{{{\rm{surf}}}}}^{\{110\}}$, USF energy, ${\gamma }_{{{{\rm{USF}}}}}^{\{112\}}$, shear modulus, *G*, misfit parameter, *δ*, and Pugh’s ratio, *G*/*B*, where *B* is the bulk modulus) and objectives (the ductility index, *D*, and CRSS, *τ*_*y*_) evaluated along the Pareto fronts (marked with blue-green for MoNbTi and with red-yellow for MoNbTiTa). **a–e** Display the correlations between the *D* and material parameters, while **f–j** show the correlation between τ_y_ and the materials parameters. MoNbTiTa’s PFA has two segments: PFA2 is represented by the predominantly yellow tail, PFA1 overlaps with the PFA of MoNbTi.

The VBA model is very efficient and accurately captures the strength and ductility parameters, enabling exhaustive scans across the entire concentration space. This allows us to track the evolution of material parameters as new alloy components are introduced and also quickly evaluate feasible regions with high resolution, which would otherwise imply significant computational costs. For example, by scanning over a dense mesh of concentrations for MoNbTi, MoNbTiTa, we can visualize the feasible regions of the two objectives (*D* and *τ*_*y*_) as functions of the most influential material properties, with the results displayed in Fig. 10. For the planar defect energies, we use the dominating set of orientations ({110}/{112}), which determines the value of *D* for most of the Pareto-optimal compositions.

Examination of the feasible regions themselves already reveals some general trends. From the shapes of the regions one can infer that, generally, the CRSS correlates with the misfit parameter, *δ*, and the shear modulus, *G*, and only weakly with the USF energy, *γ*_USF_, while possessing practically no correlation with *γ*_surf_. Interestingly, despite the considerable correlation with the shear modulus, *τ*_*y*_ exhibits no particular trends in terms of the Pugh’s ratio, *G*/*B*. At the same time, the ductility index, *D*, has clear correlations with the surface and USF energies (in the form of the reciprocal relation) and a rather strong correlation with *G*/*B*, while the correlations with *G* and *δ* are relatively weak. An important conclusion to be drawn here is that, since *τ*_*y*_ and *D* only weakly correlate with *γ*_surf_ and *δ*, respectively, the latter two material parameters offer a possibility for an independent fine-tuning of the ductility and strength.

The trends inferred from the shapes of the feasible regions are, of course, not surprising. However, the analysis of the Pareto-optimal compositions provides us with a more refined picture. Firstly, one portion of the PFA for MoNbTiTa is almost completely overlapping with the PFA for MoNbTi. We will refer to this portion as MoNbTi(Ta). This portion of the PFA of MoNbTiTa corresponds to PFA1, which was previously discussed. The other visible portion of MoNbTiTa corresponds to PFA2.

The most prominent observation, when examining the Pareto fronts, is the consistent change in trends between the two portions of the PFA of MoNbTiTa. If one Pareto front shows a positive correlation, another one shows a negative correlation. For instance, although high surface energies are generally beneficial for ductility, *D* in MoNbTi(Ta) increases (i.e., the material becomes less ductile) at higher values of *γ*_surf_, mainly because *γ*_USF_ (and *G*) increases faster. However, in the other portion of MoNbTiTa, this trend is reversed along the PFA, where the ductility improves (*D* decreases) thanks to a comparable rate of increase in both *γ*_surf_ and *γ*_USF_.

Notably, the behavior of the Pugh’s ratio, *G*/*B*, is much more non-trivial than what one could previously deduce from the plots of feasible regions. While it effectively distinguishes between brittle and ductile composition regions similar to *D*, the positive correlation with *D* is observed only along a more brittle part of the PFA for MoNbTi(Ta), while the more ductile part as well as the PFA of MoNbTiTa reveal a clear negative correlation with the ductility index. This suggests that blindly following the Pugh’s criterion for improving ductility might result in very unfavorable compromises on the strength.

Finally, another way to use Fig. 10 is to rationalize the effects of the addition of Ta already visible in the Pareto-front representation in Fig. 3. The observed increased strength of optimal compositions with Ta (along the PFA) can be primarily attributed to Ta enhancing the misfit parameter, *δ* (see also Fig. 4a), and, to a certain extent, enhancing the shear modulus *G*. However, although high strength alloys are associated with larger values for both, *G* and *δ*, the maximum of these two quantities does not necessarily mean maximum strength, as is evident from the locations of the highest values of *τ*_*y*_ at the Pareto fronts. Furthermore, the reduced ductility of Ta-containing alloys can be attributed to lower surface energies, which is not compensated by the modest decrease in USF energies. This also highlights how the Pugh’s criterion can fail to predict the correct ductility trend when the alloy surface energy is ignored.

**Fig. 11:**
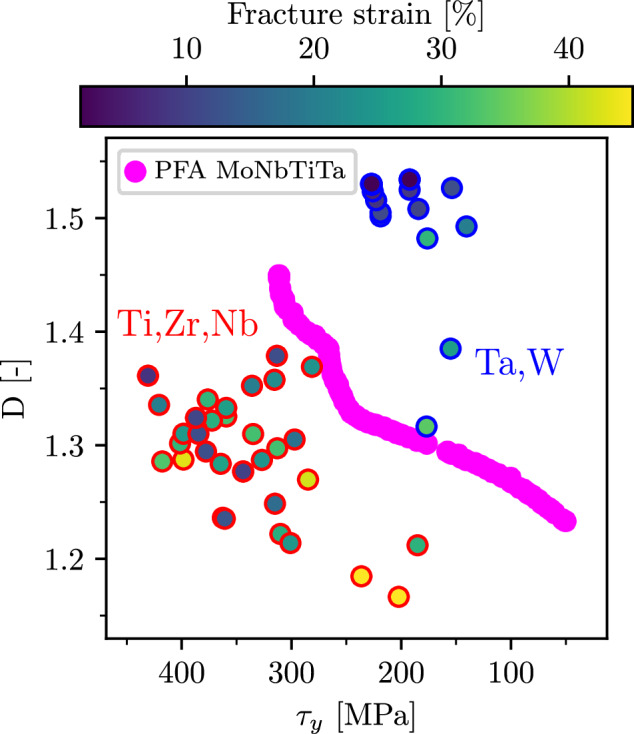
Ductility index, *D*, and CRSS, *τ*_*y*_ evaluated for various alloys using the VBA model. Red circles indicate Ti, Zr, and Nb-rich alloys, while blue circles denote Ta and W-rich alloys, with the fill color (from dark blue to yellow) corresponding to the measured fracture strain^23^. The Pareto front of MoNbTiTa is plotted in magenta points.

To check some of the above observations and to demonstrate the potential of the model for exploration of other systems, we extend our investigation of trends in *D* and *τ*_*y*_ to a wider range of alloys comprising elements Nb, Ta, Mo, W, Ti, V, Zr, and Hf. Specifically, we consider the same alloy compositions as in Fig. 8 and apply the VBA model to compute all necessary properties for the main objectives, *D* and *τ*_*y*_, with the results shown in Fig. 11. We also add values from the Pareto set of MoNbTiTa (mind the difference in scales when comparing to Fig. 3), which puts this alloy system into a broader context.

A remarkable feature of Fig. 11 is an evident separation of points into two distinct clusters. These clusters can be categorized into specific alloy groups characterized by certain primary elements. One cluster, with points marked by red circles, consists of alloys rich in Ti, Zr, and Nb, which span a range of CRSS values from 200 to 400 MPa and have moderate to high values of the fracture strain (equivalently, moderate to low values of *D*). Another cluster, with points marked by blue circles, comprises alloys rich in Ta and W and these alloys are characterized by smaller fracture strains and systematically high values of *D*. Another remarkable feature is that the Pareto front of MoNbTiTa almost perfectly separates these two clusters, suggesting that the Pareto set represents the most optimal alloys in the class of Ta- or Mo-rich alloys (and containing only moderate amount of ductilizers in the form of Ti). A more detailed analysis shows that superior properties of most of the alloys in the “red” cluster can be roughly rationalized along the lines of the conclusions we have made from Fig. 10. The *D*-parameter of these alloys is within the range of values on the Pareto front of MoNbTiTa, which also applies to the surface and USF energies. However, values of *δ* for the alloys from the cluster exceed the misfit parameter of MoNbTi by a factor of 2-4, making them much stronger and thus placing them far beyond the Pareto front of MoNbTiTa.

In conclusion, we have demonstrated how a combination of CPA and actively-learned MTPs can solve the problem of predicting the CRSS and intrinsic ductility of refractory MPEAs over the whole composition space with ab initio accuracy, a problem that is currently not tractable with conventional ab initio-based methods. In particular, by integrating our ab initio automated workflow into a robust Bayesian multi-objective optimization framework we have applied the developed approach to identifying high-performance Pareto-optimal compositions.

Furthermore, our supercell size convergence tests enabled by MTP-powered molecular statics have revealed that sizes of typical supercell sizes used in conventional SQS calculations are often insufficient for accurate calculations of certain important materials properties, such as the surface energy, of multi-component alloys. Specifically, the surface energy of MoNbTi has been shown to exhibit a rather slow convergence with the supercell size, which leads to errors of 10–20% for SQS supercells of about 100 atoms.

We have applied our multi-objective optimization framework to the four-component MoNbTiTa alloy system and exemplified its use by investigating how alloying with Ta affects the strength-vs-ductility trade-off. Interestingly, we have found that, in this particular case, instead of advancing the Pareto front of the three-component system, the addition of Ta resulted in the emergence of a separate optimal region, extending the initial Pareto front of MoNbTi to higher strength values. Such a behavior represents a peculiar case of a disjoint Pareto sets in the composition space, which might have important implications for alloy design.

Furthermore, we have used the results from the workflow to validate a model based on the virtual bond approximation (VBA), which captures the bonding mechanism of transition metals. Relying on only a couple of element-specific parameters and universal functions, our VBA model has proved to provide estimates of alloy properties over the entire concentration space that are in very good agreement with direct CPA+MTP calculations. We have, then, used the VBA model to examine the trade-off between *τ*_*y*_ and *D* in terms of relevant material parameters for a large class of refractory alloys. The results suggest that an improved trade-off in alloy design can generally be achieved if one fine-tunes the CRSS and ductility index by targeting the misfit parameter and the surface energy, respectively. This analysis also demonstrates how the VBA model can be a useful tool for examining trade-offs between competing properties in a large compositional space of refractory alloys.

Although our study focuses on strength and ductility, the proposed methodology can be readily applied to other mechanical properties, such as precipitation strengthening, which would involve interface energies or anti-phase boundary energies. Moreover, given the good accuracy of the proposed VBA model, it may also be used as a surrogate model in a multi-fidelity optimization framework, where ab initio evaluations of the objectives are triggered only rarely, allowing for efficient on-the-fly optimization of alloy properties.

Another prospective direction for development is the integration of recently developed multicomponent magnetic MTPs^83^ into the presented framework, opening the possibility to treat magnetic alloys with complex magnetic configurations. This can be also complemented by the learning approach, whereby the MLIPs are trained on the fly using fragments of the whole system^84^, which could broaden the range of applicability of our framework to systems that are, in principle, barely tractable by conventional Kohn-Sham DFT, e.g., isolated dislocations.

## Methods

### Analytical model for intrinsic ductility

To predict the intrinsic ductility of a material, the Linear Elastic Fracture Mechanics (LEFM) based theory is employed. This theory, initially introduced by Rice and Thomson^57^, has recently found application in the description of ductility of alloys and has been enhanced by incorporating atomistic insights^41,85^. It aims to describe the behavior of ideal sharp cracks in elastic bodies and evaluates a material’s propensity for failure due to crack propagation. This is done by balancing the stress intensity or strain energy release rate *K*_Ic_ and *K*_Ie_ of the governing processes. The competition between brittle cleavage and ductile dislocation emission at the crack tip determines the material’s behavior, which is reflected in the stress intensity. Plastic deformation at the crack front results in a reduction of stress intensity, which ultimately can lead to an increase the total fracture toughness. We only consider in our investigation mode *I* loading (stress orthogonal to the local plane of the crack surface), which generally limits the ductility the most. Crack geometries that dominate ductility show cleavage on the {110} or {100} planes and dislocation emission on the {110} or {112} planes based on refs. ^41,42,86,87^. We will refer to these fracture systems as {crack plane}/{emission plane}. The ratio of the *K*-factors for a given crack geometry gives the ductility index

2 $$D=\frac{{K}_{Ie}}{{K}_{Ic}},$$

where intrinsic ductility is achieved for *D* < 1 at *T* = 0*K*. The stress intensity factor *K*_Ic_ for cleavage fracture is given by

3 $${K}_{{{{\rm{Ic}}}}}=\sqrt{\frac{2{\gamma }_{{{{\rm{surf}}}}}}{{\lambda }_{22}(\underline{\underline{C}})}}$$

and stress intensity factor *K*_Ie_ for dislocation emission is given by

4 $${K}_{{{{\rm{Ie}}}}}=\frac{\sqrt{{\gamma }_{{{{\rm{USF}}}}}\,o(\underline{\underline{C}},\theta ,\phi )}}{{F}_{12}(\underline{\underline{C}},\theta )\cos (\phi )},$$

where *θ* is the angle of the slip plane with respect to the crack front, *ϕ* is the angle of the Burgers vector *b* inclined to the slip direction, *γ*_USF_ is the unstable stacking fault energy of the emission plane, *γ*_surf_ is the surface energy of the crack plane and $\underline{\underline{C}}$ is the linear elastic tensor. *λ*_22_ is Stroh’s anisotropic elasticity parameter^88^. *F*_12_ and *o* are geometrical and anisotropic parameters^85^.

### Analytical model for yield strength

The yield strength in bcc alloys with dominating solid solution strengthening can be evaluation with the model proposed by Maresca and Curtin^36,37^. Within their model, the temperature and strain-rate-depended yield stress is characterized by two thermal activation models specific to certain temperature ranges. In the low-temperature/high stress (*τ*_*y*_/*τ*_*y*0_ > 0.5) range, *τ*_*y*_(*T*) is given by,

5 $${\tau }_{y}(T)={\tau }_{y0}\left(1-{\left(\frac{kT}{\Delta {E}_{b}}\ln \frac{{\dot{\varepsilon }}_{0}}{\dot{\varepsilon }}\right)}^{\frac{2}{3}}\right),$$

whereas for higher temperatures/low stress (*τ*_*y*_/*τ*_*y*0_ > 0.5),

6 $${\tau }_{y}(T)={\tau }_{y0}\exp \left(-\frac{1}{0.55}\frac{kT}{{E}_{b}}\ln \frac{{\dot{\varepsilon }}_{0}}{\dot{\varepsilon }}\right).$$

In our calculations, we assume a $\dot{\varepsilon }$ of 5 ⋅ 10^−4^ *s*^−1^ and ${\dot{\varepsilon }}_{0}$ of 10^4^ *s*^−1^.

In the above equations, *τ*_*y*0_ and Δ*E*_*b*_ are, respectively, the zero-temperature yield stress and the activation barrier, given by the following expressions:

7 $${\tau }_{y0}=0.04{\alpha }^{-\frac{1}{3}}\overline{\mu }{\left(\frac{1+\overline{\nu }}{1-\overline{\nu }}\right)}^{\frac{4}{3}}{\delta }^{\frac{4}{3}},$$

8 $$\Delta {E}_{b}=2.00{\alpha }^{\frac{1}{3}}\overline{\mu }{\overline{b}}^{3}{\left(\frac{1+\overline{\nu }}{1-\overline{\nu }}\right)}^{\frac{2}{3}}{\delta }^{\frac{2}{3}},$$

where *α* = 1/12, $\overline{B}$, $\overline{\mu }$ and $\overline{\nu }$ are averaged bulk, shear modulus and Poisson ratio, *b* is the average Burger’s vector and the misfit parameter is $\delta =\frac{4}{3\sqrt{3}}\sqrt{{\sum }_{i}{c}_{i}{(\Delta {V}_{i})}^{2}}/(V)$. The averaged linear elastic properties are obtained by:

9 $$\overline{\mu }=\sqrt{\frac{1}{2}{C}_{44}({C}_{11}-{C}_{12})}$$

10 $$\overline{B}={C}_{11}+2{C}_{12}$$

11 $$\overline{\nu }=\frac{3\overline{B}-2\overline{\mu }}{2(3\overline{B}+\overline{\mu })}$$

The misfit volumes Δ*V*_*i*_ are obtained from a series of CPA calculations for systems with small deviations in the concentrations from the original alloy in the same way as in our earlier work^59^. For each elemental component, four calculations are performed where the concentration of the respective element is changed by − *δ*, − *δ*/2, *δ*/2, and *δ* (*δ* = 0.005) from the original alloy. The concentration ratios among the remaining constituent elements are kept constant. The linear elastic constants were obtained from volume-conserving monoclinic and orthorhombic distortions following the computational details described in refs. ^89,90^. To extract CRSS *τ*_*y*_ from experimental data of poly-crystalline samples, we subtract an estimated Hall-Petch contribution from ref. ^91^ and divide the result by the Taylor factor of 3.09.

### First-principles evaluation of materials parameters

To determine the relevant materials parameters that affect ductility and strengthening, various ab initio methods based on density-functional theory are used.

Elastic constants, equilibrium volumes and concentration derivatives of equilibrium volumes are evaluated with the exact muffin-tin orbital (EMTO)^92^ code (Lyngby version^93^) implementing a Green’s function based DFT methodology combined with CPA to perform total energy calculations. Parameters for screened Coulomb interactions in the CPA are obtained by the use of the EMTO-based locally self-consistent Green’s function technique^94,95^. Accurate total energies are obtained within the full-charge density formalism^96^. We evaluate finite-temperature equilibrium volumes following our previously suggested methodology^59^ with LDA as the reference functional for the pressure correction calculation. The elemental reference volumes are obtained by extrapolation of cryogenic lattice constant measurements^97–103^ to 0K. Zero point vibrations are included in the calculations by a quasi-harmonic Debye model *U* = 9/8*Nk*_*b*_Θ_*D*_(*V*) as described in refs. ^104,105^.

The unstable stacking fault energies, *γ*_USF_, and surface energies, *γ*_surf_, are obtained using Moment Tensor Potentials (MTPs)^53,106–108^ which are actively-learned from DFT. The total energy of MTPs is given by a sum over all *i* atoms in the configuration

12 $${E}_{{{{\rm{MTP}}}}}=\sum\limits_{i}\sum\limits_{\alpha }{\xi }_{\alpha }{B}_{\alpha }({n}_{i}),$$

where *ξ*_*α*_ are linear parameters and *B*_*α*_(*n*_*i*_) basis functions that depend on the neighborhood *n*_*i*_ within some cutoff radius around atom *i*. The scalar basis functions are obtained from contractions of the moment tensor descriptors

13 $$M_{\mu,\nu} (n_i) = \sum\limits_j f_\mu (\vert {\mathbf{r}}_{ij} \vert) \underbrace{{\mathbf{r}}_{ij} \otimes \cdots \otimes {\mathbf{r}}_{ij}}_{\nu\,{\rm{times}}},$$

where the per-atom radial functions *f*_*μ*_(∣**r**_*ij*_∣) are expanded in terms of Chebyshev polynomials. The total number of basis functions contained in *E*_MTP_ is defined by the MTP level. For the hyperparameters of the MTP, we choose a level of 16 and a cutoff of 5.0 Å.

The training sets are constructed using the algorithm proposed in^56^. Below, we highlight only the most important points of the algorithm including our small modifications. For a more detailed description we refer to the original work of Hodapp et al.^56^.

In the first step, we randomly sample a set of training candidates containing 72/96-atom surface, 54-atom bulk, and 72/96-atom unstable stacking fault configurations on a regular concentration grid within a simplex. To explore more extrapolative configurations, we also introduce slight displacements of atoms and deformations of the cells. Choosing a grid with four points in each dimensions was found to be sufficient to predict energies over the entire composition space for the initial training set (for details, see^56^). In total, our initial training set contains 146 and 377 configurations. Next, we let active learning choose the most distinct configurations from the set of training candidates (each candidate set contains around 10000 configurations), perform static DFT calculations on them and augment the interatomic potential by retraining it with these data. The search for distinct configurations is conducted using a progressively decreasing extrapolation grade, starting from 1000 and gradually reducing to 100, 10, and finally 2. We iterate multiple times, with always newly generated training candidate sets and stop when no new training configurations emerge anymore that exceeds the extrapolation grade. This procedure resulted in a training set containing 219 and 631 configurations for MoNbTi and MoNbTiTa, respectively. Finally, we run molecular dynamics simulations at 50K, 150K, and 300K, on each of the configurations from our initial training set. During each of the simulations, active learning is used to find new potentially extrapolative configurations. We remark here that running MD was crucial for the robustness of our potentials. Training only on relaxations as done in^56^ has been found to be insufficient to ensure stability of large-scale configurations during the lattice relaxation. Our final training set contains 381 and 720 configurations for MoNbTi and MoNbTiTa, respectively. The errors on the final training set for total energies are found to be 6 and 19 meV/atom, and for forces – 0.099 and 0.104 eV/Å for the MoNbTi and MoNbTiTa potentials, respectively. A detailed performance check can be found in Supplementary Information.

The final properties (unstable stacking fault and surface energies) are then calculated on a cell with about 300000-500000 atoms to include far-field effects and to ensure proper statistics. We provide a detailed convergence study for the cell size in Supplementary Information.

The DFT computations for the MTP training are performed with the Vienna Ab-initio Package (VASP)^109–111^ using the PBE functional^112^ with an energy cutoff of 400 eV. We use a Monkhorst-Pack k-point mesh with a spacing of 0.15 Å^−1^. Moreover, we use Gaussian smearing with a smearing width of 0.08 eV to avoid potential problems of negative occupations. The standard pseudopotentials (PP) were used for Mo, Ti, and Ta^113,114^. For Nb, PP including semicore p-states as valence states was used.

### Multi-objective Bayesian-optimization

In order to effectively find the Pareto front of our multi-objective Bayesian optimization problem, we have adapted a methodology developed by Galuzio et al.^115^. This method involves generating a Pareto front approximation (PFA) from an easy-to-evaluate surrogate model at each search step, and selecting the next evaluation point from this PFA before updating the surrogate model. This iterative process enables the PFA to gradually approach the true Pareto front. We can also narrow the search space by excluding compositions that are thermodynamically unstable or possess unfavorable properties.

To navigate through the search space, we employ the following sequential design strategies:

1. We begin the search process by building a smooth surrogate model. To that end, we are using Gaussian processes regression (GPR) based on the observations available from previous rounds. For the initial round, we sample a coarse regular grid in the search space. The choice of the covariance function $K({{{\bf{x}}}},{{{{\bf{x}}}}}^{{\prime} })$ is determined using cross-validation on grid test sets. We have selected a sum of white noise kernels and radial basis function kernels as the best:

14 $$K({{{\bf{x}}}},{{{{\bf{x}}}}}^{{\prime} })={\sigma }^{2}{{{\bf{I}}}}+C\exp \left(-\gamma {\left\Vert {{{\bf{x}}}}-{{{{\bf{x}}}}}^{{\prime} }\right\Vert }^{2}\right),$$

where *σ*, *C*, *λ* are hyper-parameters estimated by maximizing the marginal likelihood on the training data.

The surrogate models for our objectives, namely the ductility index *D* and the yield strength *τ*_*y*_, are then used to make predictions at unobserved areas of the search space and quantify the uncertainty around them. This information is crucial for guiding the search towards promising areas in the search space.

2. Next, we use the non-dominated sorting genetic algorithm II (NSGAII)^58^ implemented within the DEAP library^116^ in order to identify 100 PFA points Ω_PFA_ using our surrogate model. A penalty function is used to handle constraints, such as limiting the search space and keeping it within a certain concentration range.

3. Select the next search points **x**^*n*+1^ from the current PFA, ${{{{\bf{x}}}}}^{n+1}={{{{\bf{x}}}}}_{{t}_{{{{\rm{next}}}}}}^{* }\in {\Omega }_{{{{\rm{PFA}}}}}$, that are farthermost from all previous sampling points according to

15 $${t}_{{{{\rm{next}}}}}=\,{{\mbox{argmax}}}\,\left(q\frac{{d}_{f}^{(i)}-{\mu }_{f}}{{\sigma }_{f}}+(1-q)\frac{{d}_{x}^{(i)}-{\mu }_{x}}{{\sigma }_{x}}\right),$$

where ${d}_{\{\cdot \}}^{(i)}$ corresponds to the smallest distance from the *i*^th^ point in Ω_*PFA*_ to all other points found by the algorithm. The subscript “f” indicates the distance calculated in objective space, while the subscript “x” indicates the distance in the search space. The distances are standardized by *μ*_{⋅}_, representing the average, and *σ*_{⋅}_, representing the standard deviation, of the respective distance measure. We choose the weighting parameter *q* as 0.8 to slightly favor objective space exploration.

4. Check the stopping criterion: If the maximum number of iterations has been reached or only minimal Pareto-improvement is achieved, stop the algorithm and output the Pareto front and the Pareto set. The stationarity condition on the Pareto front measures Pareto-improvement by determining how much the front changes with more points. If there is little change after several iterations, the loop terminates. Otherwise, go back to step 3 and continue the search. The final Pareto set can be discontinuous and portion of the Pareto set can be located in different regions of the concentration space. To identify separate portions, we use multivariate Gaussian mixture models to perform automatic spatial clustering and label the corresponding parts at the Pareto front. For MoNbTi, 36 points are required, whereas for MoNbTiTa, 143 points are needed, with 72 for set 1 and 71 for set 2.

### Dimensionality reduction and visualization

We employed a t-Distributed Stochastic Neighbor Embedding (*t*-SNE), a dimensionality reduction technique, to visualize the 4-dimensional concentration space in two dimensions. This projection allows us to visualize our objectives and gain insights into the overall landscape. The biggest advantage of t-SNE is that it can capture much of the high-dimensional data’s local structure while also revealing global structure. Furthermore, it is a non-linear method that can also capture the structure of complex manifolds. However, *t*-SNE provides only an approximate preservation of local structure and distances, which leads to the emergence of an observed patchy pattern in our case. The corner points of the projection correspond to the elemental compounds, the edges represent binary compositions, and the center represents the equimolar compound. The distance from the corner points in the projection is approximately proportional to the effective composition. The t-SNE projections displayed in Fig. 3 in the main text are performed from the entire concentration space, with points evaluated using the Gaussian process surrogate models for the ductility index and the strength.

### Virtual bond approximation model

Within the VBA, to second order in inter-component interactions, the energy of an alloy system can be expressed as follows:

16 $$\begin{array}{l}E(\{{c}_{i}\},\lambda )\,=\,\sum\limits_{i}{c}_{i}\,{w}_{i}{e}^{(1)}({N}_{i},\lambda )+\\\qquad\qquad\qquad+\sum\limits_{ij}{c}_{i}{c}_{j}\,{w}_{ij}{e}^{(2)}({N}_{ij},\lambda ),\end{array}$$

where *c*_*i*_ are atomic fractions of components, *e*^(*k*)^ are the virtual bond functions of the average *d*-valences, *N*^(*k*)^ ≡ {*N*_*i*_, *N*_*ij*_}, and of bandwidth parameters *w*^(*k*)^ ≡ {*w*_*i*_, *w*_*ij*_}. Here *N*_*i*_ and *w*_*i*_ refer to a respective elemental value, while second-order parameters are defined as *N*_*ij*_ = (*N*_*i*_ + *N*_*j*_)/2 and ${w}_{ij}=\sqrt{{w}_{i}{w}_{j}}$, where the latter expression is inspired by the tight-binding description of transition-metal alloys^117^. *λ* represents all other parameters, such as temperature, volume, lattice vectors, etc. The bandwidth parameters were taken from ref. ^118^ and adjusted by a small correction depending on the row of an element. Alternatively, one can fit these parameters to elemental compounds and a few binary alloys but usually the relative values obtained this way are similar to those from the above reference. Note also that the expression *E*({*c*_*i*_}, *λ*) can represent either the Gibbs free energy, *G*({*c*_*i*_}, *P*, *T*), or the Helmholtz free energy, *F*({*c*_*i*_}, *V*, *T*), where additional parameters (e.g., lattice vectors) are implied. Considering derivatives of the thermodynamic potentials with respect to various parameters, we can get VBA representations of any property that can be derived from the free energy.

From the Gibbs free energy, we can the derive an expression for the volume, *V*({*c*_*i*_}) = ∂*G*({*c*_*i*_}, *P*)/∂*P*∣_*P*=0_, omitting the temperature dependence for simplicity. Similarly, we can obtain an expression for the bulk modulus and general elastic (isothermal) constants by *B* = *V* ∂^2^*F*({*c*_*i*_}, *V*)/∂*V*^2^ and ${C}_{\alpha \beta }=\frac{{\partial }^{2}F(\{{c}_{i}\},V)}{\partial {\epsilon }_{\alpha }\partial {\epsilon }_{\beta }}$, where *ϵ*_*α*_ is the strain in Voigt notation. In the context of surface and USF energies, the molar energy difference, Δ*E* = *E*_defect_ − *E*_bulk_, between the defective and bulk structures is used

17 $$\begin{array}{l}{{{\boldsymbol{\xi }}}}(\{{c}_{i}\})\,=\,\sum\limits_{i}{c}_{i}\,{w}_{i}\,{{{{\boldsymbol{\xi }}}}}^{(1)}\left({N}_{i}\right)+\\\qquad\qquad+\sum\limits_{ij}{c}_{i}\,{c}_{j}\,{w}_{ij}\,{{{{\boldsymbol{\xi }}}}}^{(2)}\left({N}_{ij}\right),\end{array}$$

where **ξ**({*c*_*i*_}) is one of *B*, ${C}^{{\prime} }$, *C*_44_, *V*, or Δ*E*, and **ξ**^(*k*)^ represent the corresponding virtual bond functions. To obtain the unknown **ξ**^(*k*)^, we perform a series of calculations of the associated quantity for several systems (elemental compounds and selected binary, three- and four-component alloys). The unknown values of virtual bond functions at integer (for *ξ*^(1)^) or half-integer values (for *ξ*^(2)^) are then obtained by means of linear regression from the above equation, since the values of *c*_*i*_ and *w*_*i*_ are known for each composition. The discrete function values obtained in this manner for our refractory alloys behave very well and can be represented as values of smooth functions of valences. In fact, we use second and third-order polynomials to accurately represent, respectively, the first order virtual bond function **ξ**^(1)^ and the second order virtual bond function **ξ**^(2)^ as a way of conveniently obtaining a value for a given *N*. This implies that virtual bond functions can be used to consistently characterize the system. An example of the virtual bond function can be found in Supplementary Information.

The material parameters for determining the corresponding virtual bond functions have been obtained using CPA calculations. For planar defects we first calculate *unrelaxed* surface and USF energies with CPA for elemental compounds and binary alloys and parameterize the VBA model, as described above. We have checked that the VBA model predicts the CPA values very well (see Supplementary Information). To account for relaxation effects, we simply adjust the virtual bond functions to align with the *relaxed* surface and USF energies of individual elemental compounds. More precisely, we scale the virtual bond functions by a factor that follows a linear relationship with the *d*-valences. Such an approach works because fitting to CPA values already includes most of the chemical interactions and the strongest relaxation effects have geometrical nature, which can be taken into account by the rescaling procedure of the universal virtual bond functions.

### Supplementary information

Suplementary Information

## Data Availability

The optimization results for MoNbTi and MoNbTiTa, MTP potentials including their configuration files, and the virtual bond approximation model are all available at Zenodo under 10.5281/zenodo.12516334.

## Code availability

The codes used to carry out this work are described and referenced in the Methods section and are available free-of-charge with the exception of VASP. The software utilized in this study are accesible at: MTP-2 https://gitlab.com/ashapeev/mlip-2, LAMMPS https://github.com/lammps/lammps, ATAT v3.6 https://www.brown.edu/Departments/Engineering/Labs/avdw/atat/. Additionally, EMTO, ELSGF, mtp4py, and the automated workflows are accessible upon reasonable request from the authors.
