# Supplementary material for: Ab initio framework for deciphering trade-off relationships in multi-component alloys
Source: NPJ Comput Mater. 2024 Jul 16;10(1):152. doi: 10.1038/s41524-024-01342-2 (PMC11275555; doi:10.1038/s41524-024-01342-2)
Supplement: Supplementary file 1 — Suplementary Information [file 41524_2024_1342_MOESM1_ESM.pdf]

Supplementary Information: Ab initio framework for deciphering trade-off relationships in multi-component alloys

Franco Moitzi [1*], Lorenz Romaner [2], Andrei V. Ruban [1,3], Max Hodapp [1], Oleg E. Peil [1]

[1*]Materials Center Leoben Forschung GmbH, Roseggerstraße 12, Leoben, A-8700, Austria.
[2]Chair of Physical Metallurgy and Metallic Materials, Department of Materials Science, University of Leoben, Roseggerstraße 12, Leoben, A-8700, Austria.
[3]Department of Materials Science and Engineering, Royal Institute of Technology, 10044 Stockholm, Sweden.

*Corresponding author(s). E-mail(s): moitzi.francopeter@gmail.com;
Contributing authors: lorenz.romaner@unileoben.ac.at; a.v.ruban@gmail.com; maxludwig.hodapp@mcl.at; oleg.peil@gmail.com;

1 Validation of phase stability of solute solution

To assess the stability and formation of solute solutions and the occurrence of competing intermetallic phases, various approaches can be employed.

One approach involves CALPHAD utilizing thermodynamic databases, albeit constrained by the availability and also accessibility of such databases. Alternatively, there are also fully ab initio-based approaches to evaluate phase stability, although they tend to be computationally too expensive and complex to be utilized inside an automated workflow. Accurate and reliable prediction of phase transformations can be a non-trivial task even for binary systems.

A more straightforward strategy involves assessing simpler quantities such as the formation energies of solid solutions and ordered phases, complemented by experimental observations to establish criteria for estimating phase stability.

We employ the formation energies criterion from Ref. [2] in our work to evaluate operational stability of our alloy and to set *a priori* boundaries of the design space. This criterion relies on the observation that multiple phases form when $E_f > 70\,\mathrm{meV}$ and metastable metallic glasses form or ordered phases appear when $E_f < -150\,\mathrm{meV}$ [3]. In our case, the formation energies of the entire system fall within these boundaries, as illustrated in Supplementary Fig. 1 and Supplementary Fig. 2.

Furthermore, we also take into account the ordering energies for binaries on or near the convex hull of formation energies. The ordered phases were obtained from supplementary materials of Zheng *et al.* [1] and materialsproject.org. The ordering energies are determined by subtracting the formation energy of the ordered intermetallic phase from the formation energy of the solid solution, $E_{\mathrm{ord}} = E^{(\mathrm{SS})}_{f,BCC} - E^{(\mathrm{I})}_{f,BCC}$.

In Supplementary Fig. 1, it is evident that only the MoNb binary side exhibits a significant ordering energy, peaking at around 80 meV. In the four-component system, slightly higher ordering energies are observed at the TaMo binary side, reaching 120 meV. However, this corresponds only to a small region close to the boundary of the

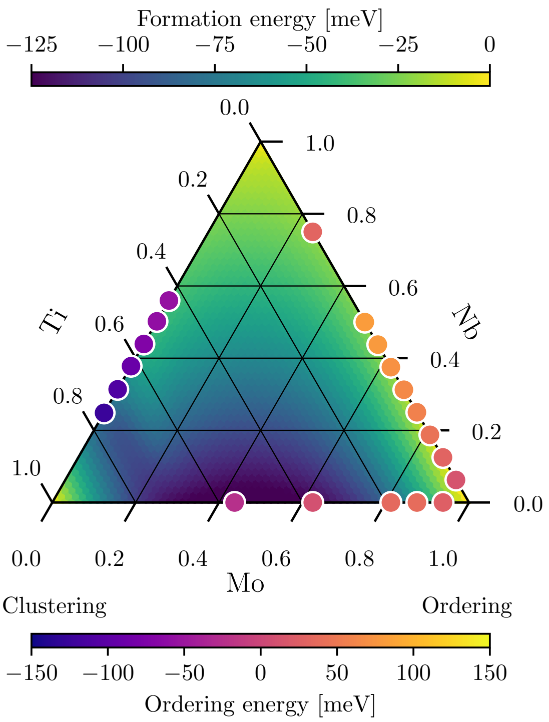

Supplementary Figure 1 Ternary plot showing a heatmap of the formation energies of the MoNbTi solid solution. Dots denote binary compositions corresponding to intermetallic compounds on the convex hull from Ref. Zheng *et al.* [1], with the fill color indicating the respective ordering energies.

concentration space, which is irrelevant for the optimization. Moreover, in a multicomponent alloy, nucleation of an ordered phase consisting of two elements can be hindered by interactions between these two elements and other components.

Most importantly, experimental evidence strongly supports the assumption that MoNbTi and MoNbTiTa form solid solutions. Mo, Nb, and Ta are fully miscible at typical annealing temperatures and readily form a solid solution. Various experimental studies have demonstrated that the addition of Ti to the MoNbTa system also leads to the formation of a BCC solid solution [4–6].

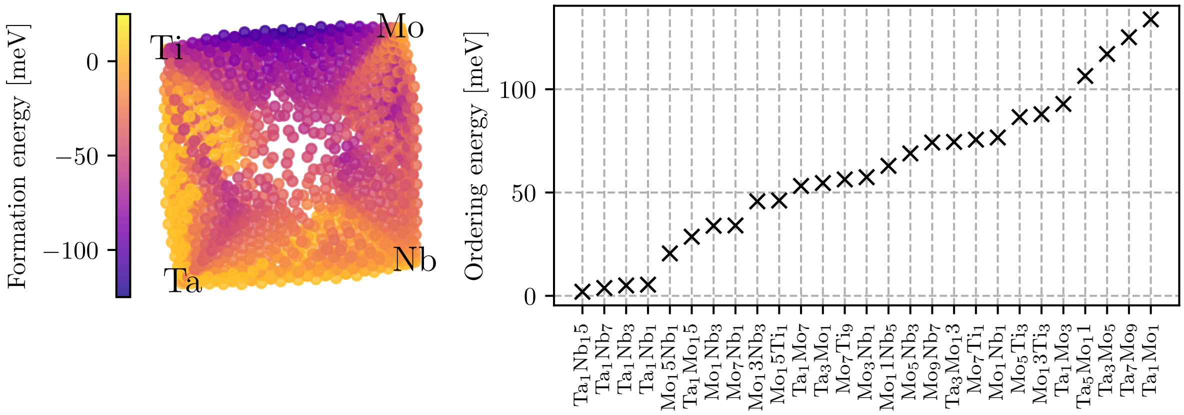

Supplementary Figure 2 (upper panel) t-SNE projection of the formation energies of the MoNbTiTa system and (lower panel) positive ordering energies binaries.

2 Validation of mechanical parameters

2.1 Validation of Ductility calculations

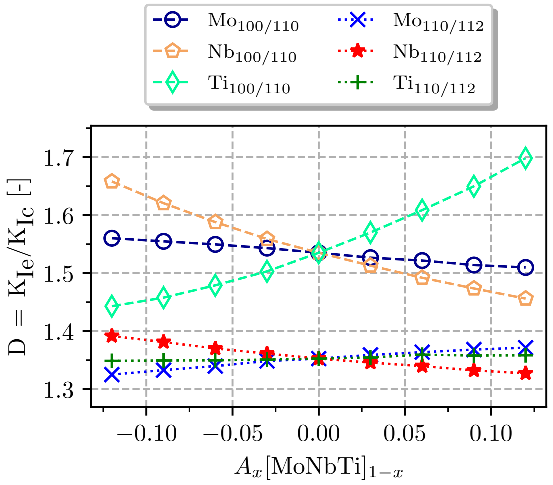

Supplementary Figure 3 Comparison of the ductility index D for the different fracture systems 100/110 and 110/112 (denoted by subscript) upon changing the concentration of one component in the equimolar MoNbTi alloy according to $A_x[\mathrm{MoNbTi}]_{1-x}$, where x is the molar fraction added to the equimolar alloy.

Experimental verification of the ductility model for a variety of refractory MPEAs has been performed in Ref. [7]. Following the same approach, we compute the ductility index, D, as a minimum of two values, $D_{100/110}$ and $D_{110/112}$, corresponding to two surface orientations, $\{100\}$ and $\{110\}$, and two stacking fault orientations, $\{110\}$ and $\{112\}$, respectively. USF, γ_{USF}, and the surface energy, γ_{surf}, is obtained from molecular statics of fully relaxed structures using MTPs. Unlike the SQS approach, which requires full structure relaxation calculations for each property, crystallographic orientation, and composition in a cell with typically 800-1000 atoms for a three-component alloy, our active-learning approach generates 381 and 720 *static* configurations with 54-96 atoms/configuration to fit an accurate potential in the entire composition space of MoNbTi and MoNbTiTa, respectively.

Using the USF and surface energies, along with the linear elastic parameters from CPA, we evaluate parameters $D_{100/110}$ and $D_{110/112}$ and ultimately the ductility index, D. Our values of the ductility parameters, $D_{100/110} = 1.53$ and $D_{110/112} = 1.35$, in the equimolar MoNbTi, corresponding to $x = 0$ in Supplementary Fig. 3, are somewhat different then the ones (1.29 and 1.59, respectively) reported in Mak et al. [7]. This can be attributed to supercell size convergence issues associated with SQS surface energy calculations. In Supplementary Fig. 3, we examine D for the MoNbTi alloy as a function of varying concentrations of each component $A \in \{\mathrm{Mo}, \mathrm{Nb}, \mathrm{Ti}\}$ in the two fracture systems, 100/110 and 110/112. The concentration of A is varied, keeping the proportion between the rest of the alloy equal, i.e., the general formula of a modified alloy is $A_x[\mathrm{MoNbTi}]_{1-x}$. Notably, the 110/112 fracture system is the dominating one across all cases, as it has the lowest D index.

Increasing Nb content enhances ductility, while Mo and Ti additions show no positive effect. Nb's softening properties are experimentally documented in Refs. [8–10].

2.2 Validation of solid solution strengthening calculations

Maresca *et al.* [11] suggested that in concentrated alloys, especially at high temperatures, solid solution strengthening can be described based on the alloy's average volume, linear elastic moduli, and misfit volumes of components. In particular, the determination of linear elastic constants and concentration derivatives of equilibrium volumes becomes challenging when employing supercell methods such as Special Quasirandom Structures (SQS). The presence of broken symmetry in SQS introduces direction-induced errors in elastic constants that are challenging to alleviate [12]. Additionally, obtaining concentration derivatives poses difficulties, as SQS is not effective for arbitrary concentrations. All of these parameters can be obtained robustly within CPA [13–15] because local atomic relaxations have a minor effect.

Given the computational efficiency of CPA it eliminates the need for extrapolative models for misfit volumes, distinguishing it from prior studies [16, 17]. While misfit volumes often exhibit a linear dependence on concentration in refractory

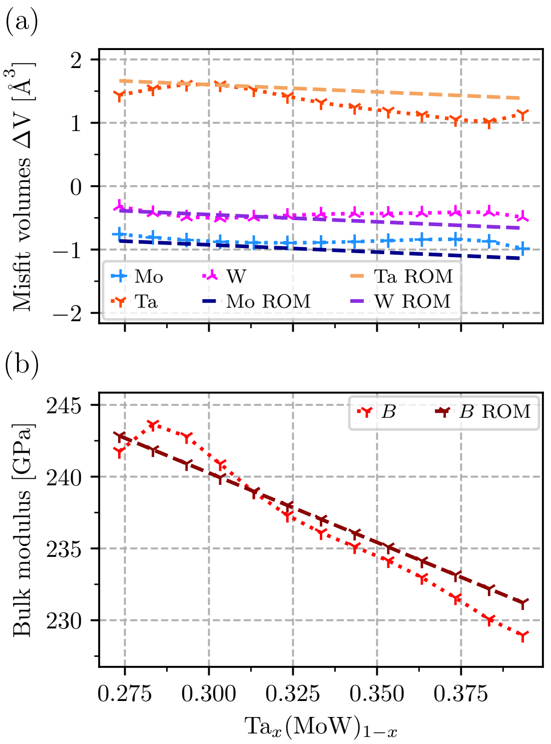

Supplementary Figure 4 Comparison of directly calculated and values from the rule-of-mixture for (a) misfit volumes and (b) bulk modulus of a MoTaW alloy with respect to Ta variation.

alloys, our research identifies specific alloys where this relationship deviates notably. Supplementary Fig. 4 illustrates the misfit volumes and bulk modulus that were directly calculated alongside those determined using ROM for variations of Ta within the MoTaW alloy. Similarly, misfit volumes and bulk modulus shows quite significant deviations form the linear trends. Especially around 0.28 the bulk modulus abruptly changes, which can be attributed to topological transitions. In Fig. 4b, we find good agreement between our CPA results and those obtained from an embedded-atom potential for A-atom in larger cells, as detailed in Refs. [18–20].

To verify the final accuracy of the strengthening calculations, we compared the accuracy of our CRSS values calculated at 300 K to room-temperature (RT) experimental data from tensile tests [21, 22], as well as compression tests reported in Ref. [23]. The poly-crystalline samples used in the experiments were reported to be single-phase. To extract the CRSS from these tests, we subtract the estimated Hall-Petch contribution of 20 MPa [24] and divide the result by the Taylor factor of 3.09. In comparison to the experimentally measured total yield strengths, the strengthening contribution of the grain boundary is small.

However, for NbTaTi, the deviations are quite large. Our theoretical τ_y value for NbTaTi is approximately 88 MPa, which is about half of the experimental value obtained from tensile tests, which yields a CRSS of around 168 MPa. To elucidate the nature of this discrepancy, we have performed a comprehensive analysis of solid solution strengthening in five additional 3- and 4-component MPEAs: NbTiZr, MoNbTi, CrMoTaTi, MoNbTaW and MoTaW. Among the alloys tested, NbTiZr, MoNbTi, and MoNbTaW exhibit similar τ_y values of around 300 MPa in experiments, whereas our theoretical approach yields 293, 195, and 234 MPa, respectively. The 4-component CrMoTaTi alloy, which exhibits a yield strength of 621 MPa in experiments, displays close agreement with our theoretical prediction of 577 MPa.

By comparing Fig. 4a with Fig. 4c, one can see that the theoretical predictions aligns closely with experimental data when the average misfit δ is high, as exemplified by NbTiZr and CrMoTaTi. All other alloys have smaller misfit deltas and their theoretical predictions consistently underestimate strength. Furthermore, it seems that the relative error is reduce for stiffer alloys with larger elastic moduli.

Calculating CRSS in bcc crystals is challenging due to multiple active glide mechanisms. According to Maresca *et al.* [11, 25], the solid solution strength of our multi-component refractory metal alloys is to large extend influenced by interactions between edge dislocations and solutes, with a lesser contribution from solute-screw dislocation interactions. Building on that strengthening theory, Baruffi *et al.* [26] discovered that as the misfit parameter δ or elastic moduli increase, the dominance of edge-controlled contributions becomes more pronounced, eventually determining the strength completely. Within the used analytic strengthening model, we, however, only consider the edge-controlled strengthening. The contributions arising from screw dislocation are omitted, because their contributions are very challenging to calculate. The negative example of

NbTaTi has a relatively small misfit parameter δ and is also soft. As a result, we underestimate the strength because the screw-controlled contribution is significant and can actually not be neglected. In contrast, NbTiZr and CrMoTaTi are well-predicted because the screw contribution can be effectively disregarded. We want to emphasize, however, that as per Maresca's findings [11, 25], the edge-controlled contribution tends to become increasingly dominant at elevated temperatures. Given that refractory alloys are primarily intended for high-temperature applications, this observation strengthens the validity of using a model for strengthening that is solely based on edge contributions for all alloys.

Even though we only consider a portion of the strengthening contribution, the findings presented in Fig. 4c demonstrate a high level of consistency between the experimentally determined CRSS and our calculated values. Overall, we tend to underestimate the strength of all the alloys tested, as expected, because we ignore the screw contributions. This also implies that, we can use the values for a conservative estimate of strength. In general, the relative changes in strength that occur during alloying are accurately reproduced. We can distinguish a weaker and a stronger alloy. For example, hardness screening of Nb in MoNbTaW revealed only a modest strength increase of about 10% [27] from equimolar MoNbTaW to MoTaW (no reliable yield strength measurements are available for MoTaW). Our methodology predicts a similar magnitude of increase, from 234 MPa to 257 MPa.

3 Validation of material parameters

3.1 Convergence of local relaxation on surface and stacking fault energy

Interatomic potentials have the advantage of allowing for larger cell sizes compared to SQS methods with. Using large cells is especially important when studying far-reaching elastic effects that cannot be accurately captured in small cells or need some complex compensation procedures. In the case of surface energies, the defect structure was created by introducing a vacuum of 20 Å. We checked that fixing atomic positions of certain deep internal layers had no effect. For both surface orientation, we create orthogonal cells. In case of USF, we created a defect structure with two stacking faults in cell by simply shifting half of the atoms in $\frac{1}{4}a[111]$. We keep the cell orthogonal. We only allow for atomic relaxation normal to the fault plant and do not change the out-of-plane lattice dimension. By using large enough structures with more than 100 layers, we can account for inelastic displacement associated with the fault. This is in contrast to [7, 28].

In order to assess the necessary cell size for the calculation of surface energies, we conducted convergence tests. Fig. 9 displays the surface energies for the 100 orientation as a function of the number of layers perpendicular to the fault. Convergence varied by alloy, and MoNbTi exhibited the slowest convergence. Approximately 100 layers were sufficient for convergence for the more sparsely-packed 100 layer in the case of MoNbTi. Only about 50 110 layers are required to achieve convergence. The other alloys, MoNb and MoNbTa, require 8-10 layers to achieve convergence.

On the other hand, the necessary cell size for unstable stacking fault converges much faster, even after only a few layers. We want to stress that we don't perform optimization of the neighboring cluster vector. Our big cell will contain statistical clustering of atoms of a specific kind. We assume that by randomly distributing the atoms in the cell in a sufficiently large cell size, we can generate a truely random alloy. For comparison, we also performed reference calculations with 4x4x10 SQS cells with 160 atoms with cluster vectors closely resembling those of truly random alloys. Except for MoNbTi, the energies of SQS cells are numerically equivalent to those of large cells. Numerical errors related to the interatomic potential can be assumed to be below 5% [29].

3.2 Effects of local relaxation on surface and stacking fault energy

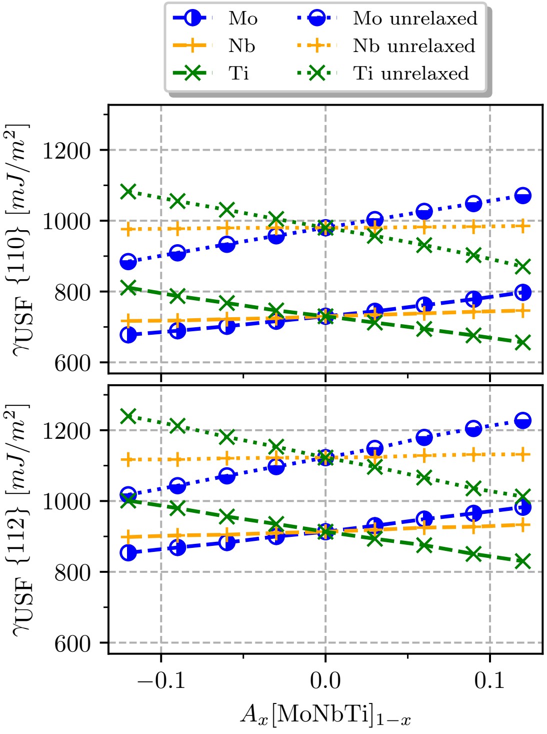

Supplementary Figure 5 Comparison stacking fault energy {110} and {112} with and without local relaxations upon changing the concentration of one component in the equimolar MoNbTi alloy according to $x\,\mathrm{Y} \rightarrow$ MoNbTi, where x is the molar fraction of Y added to the equimolar alloy.

In order to investigate the impact of local relaxation on surface and unstable stacking fault energies, we present a comparison of different compositions of the MoNbTi alloy in Figs. 5 and 6. Each line shows the change in one alloy component in relation to the equimolar composition, while keeping the ratios of the remaining components constant. Neglecting the effects of local relaxation on USF γ_{USF} yields a consistent increase of about 25 % for both orientations. The

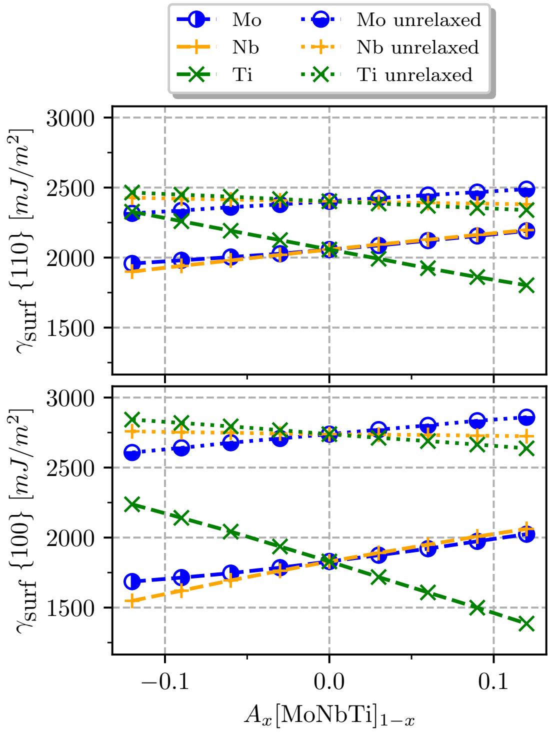

Supplementary Figure 6 Comparison surface energy {110} and {100} with and without local relaxations upon changing the concentration of one component in the equimolar MoNbTi alloy according to $x\,\text{Y} \rightarrow$ MoNbTi, where x is the molar fraction of Y added to the equimolar alloy.

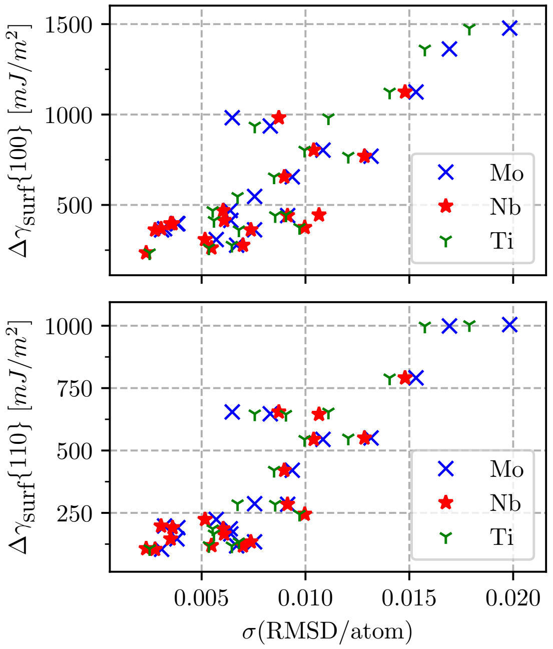

Supplementary Figure 7 Energy difference of unrelaxed and relaxed surface energies and standard deviation of RMSD value from polyhedra template matching for different compositions of MoNbTi.

overall trends of $\gamma_{\rm USF}$ remain unchanged upon changes in composition. For surface energies $\gamma_{\rm surf}$, the impact of relaxation is significant and varies depending on the element in question. Without considering relaxation effects, surface energies are largely unaffected by the addition of Nb. However, when relaxation effects are taken into account, the addition of Nb causes a significant increase in $\gamma_{\rm surf}$. Similarly, changes in Ti concentration lead to more pronounced effects on $\gamma_{\rm surf}$. On the other hand, for Mo, there is an almost constant shift between the relaxed and unrelaxed cases.

To show the origin of this behaviour, we used the Polyhedral Template Matching (PTM) [30] method to identify the local crystalline structure in order to relate this relaxation contribution to local structural changes. It also provides for each atom in the structure a RMSD value, which is a measure of the spatial deviation from the ideal local structure. The distribution of the RMSD value for each atomic species in the structure is Gaussian-like. Especially Ti showed higher local distortions compared to all other components in all alloys investigated. We also found that the mean and standard deviation of the RMSD correlate to the energy difference between unrelaxed and relaxed surface energies. The higher the standard deviation of these RMSD values, as seen in Supplementary Fig. 7, the more significant the relaxation contribution will be. The overall effect of this local structural changes, seems to be less relevant for USF.

3.3 Performance metric for MTPs

The surface and unstable stacking fault energies are relatively small and sensitive quantities. Even minor errors can have significantly impact on the final results. Therefore, simply providing errors on forces and total energies calculated with MTP for target structures may be inadequate to demonstrate the accuracy of the potential for a particular property. To verify accuracy of $\gamma_{\rm surf}$

and γ_{USF}, we generate random structures of surface and unstable stacking fault configurations, along with their respective bulk reference configurations. Atomic species are placed randomly on lattice sites for various alloy compositions and the atomic positions and the cell volumes are slightly randomly perturbed. Atomic relaxation is then performed using our MTPs. The reference energies are then computed on the relaxed structures using DFT. Across the test set, the mean absolute difference between the MTP and DFT forces is found to be 0.0402 eV/Å. Additionally, we provide a parity plot in Supplementary Fig. 8 to compare the MTP and DFT surface and unstable stacking fault energies. Overall, there is a high degree of correlation between MTP and DFT proving the accuracy of our MTPs.

3.4 Influence of the elemental distribution

In order to estimate the influence of the elemental distribution, we investigate the distributions of surface γ_{surf} and unstable stacking fault energies γ_{USF} using atomic configurations with different distributions of atomic species. To that end, we generated several hundred configurations for MoNbTi with fully random atomic distribution on the lattice sites, and using SQS. For SQS, we optimized the pair correlations of the first four shells, and the first triplet cluster correlations. We perform the optimization of the atomic configurations on the bulk structures and neglect 2-dimensional correlation at the defects. Supplementary Fig. 9 illustrates the expected narrowing of energy spread with increasing cell sizes. The standard deviation in the energies is smaller for the unstable stacking faults than for surface energies. Furthermore, the energy distribution of the SQS configuration for the stacking faults show a similar standard deviation to that of substantial bigger cell of around 10000 atoms with a purely random atom distribution. This result is in agreement with the distributions reported in the supplementary material of [1], who also used MTPs.

3.5 Error propagation

Two primary error sources arise: first, from representing a truly random alloy within a finite cell, accounting for variations in atomic configurations; and second, from approximations made by the potential.

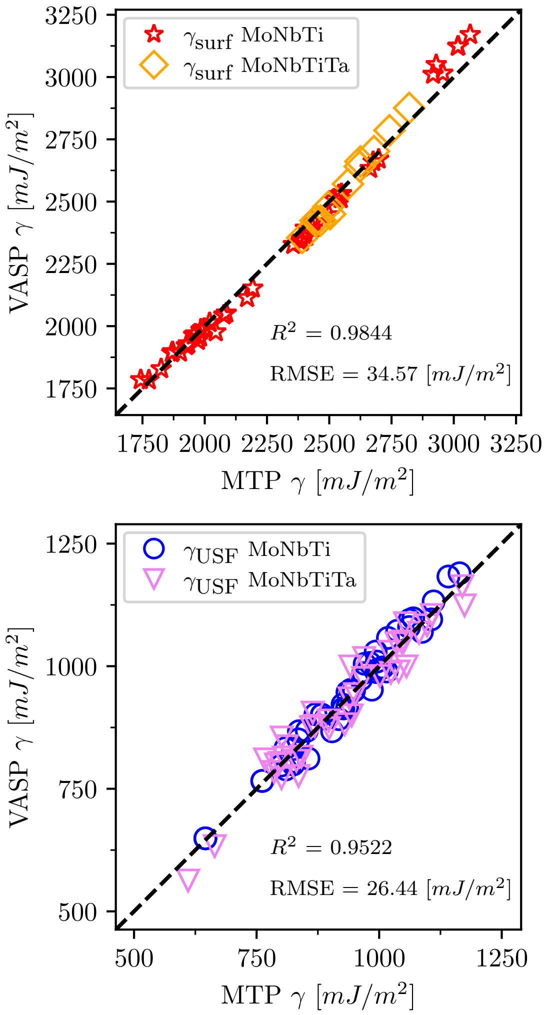

Supplementary Figure 8 Parity plot of surface and unstable fault energies obtained from MTP and directly from VASP DFT code.

From Supplementary Fig. 9 we deduce that the distributions for the surface and unstable stacking fault energies due to different atomic configurations are close to Gaussian distribution. Hence, we can perform an analytic Gaussian error propagation to estimate the final error on the D parameter,

$$D = \frac{K_{Ie}}{K_{Ic}}$$

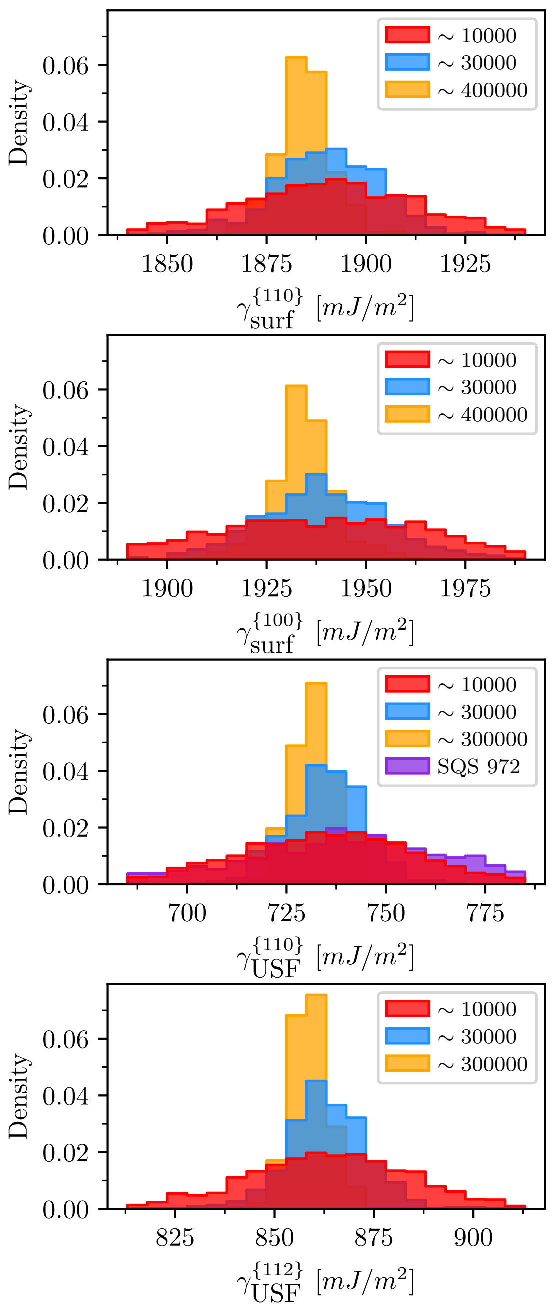

Supplementary Figure 9 Histograms of surface γ_{surf} and unstable stacking fault energies γ_{USF} for purely random configurations of different number of atoms and SQS generated cells obtained from MTPs of a near equimolar MoNbTi alloy.

$$= \frac{\sqrt{\gamma_{\text{USF}}\, o(\underline{\underline{C}}, \theta, \phi)\, \lambda_{22(\underline{\underline{C}})}}}{F_{12}(\underline{\underline{C}}, \theta) \cos(\phi) \sqrt{2\gamma_{\text{surf}}}}$$

$$= \chi \sqrt{\frac{\gamma_{\text{USF}}}{\gamma_{\text{surf}}}}.$$

Let us denote the errors as δD, $\delta\gamma_{\text{USF}}$, and $\delta\gamma_{\text{surf}}$. Since χ is computed from CPA values, it is not subjected to statistical errors and can be treated as a constant. Assuming statistical independence of $\delta\gamma_{\text{USF}}$ and $\delta\gamma_{\text{surf}}$, we have

$$(\delta D)^2 = \left(\frac{\partial D}{\partial \gamma_{\text{USF}}} \delta\gamma_{\text{USF}} \right)^2 + \left(\frac{\partial D}{\partial \gamma_{\text{surf}}} \delta\gamma_{\text{surf}} \right)^2,$$

with the partial derivatives,

$$\frac{\partial D}{\partial \gamma_{\text{USF}}} = \frac{\chi}{2\sqrt{\gamma_{\text{surf}}\gamma_{\text{USF}}}},$$
$$\frac{\partial D}{\partial \gamma_{\text{surf}}} = -\frac{\chi}{2\sqrt{\gamma_{\text{surf}}\gamma_{\text{USF}}}} \times \frac{\gamma_{\text{USF}}}{\gamma_{\text{surf}}}.$$

So, the error in D can be written as

$$\delta D = \frac{\chi}{2\sqrt{\gamma_{\text{surf}}\gamma_{\text{USF}}}} \sqrt{(\delta\gamma_{\text{USF}})^2 + \left(\frac{\gamma_{\text{USF}}}{\gamma_{\text{surf}}} \delta\gamma_{\text{surf}} \right)^2}.$$

Using the expectation values and standard deviations for the largest cell size, we obtain a value for D of 1.35 ± 0.004. Hence, we conclude that taking a large enough cell size is sufficient to practically *eliminate any influence of the elemental distribution on D*.

Obtaining the second type of error, originating from the potential, is more challenging due to limitations imposed by the size of the test set. If one were to consider the Root Mean Square Error (RMSE) values from Supplementary Fig. 8 as error estimates and assume complete independence of the errors, a straightforward calculation would yield an error of $\delta D = 0.02$. This value is approximately five times larger than the influence of the atomic contribution. However, it's essential to acknowledge that the errors stemming from the potentials of γ_{USF} and γ_{surf} are, in fact, correlated.

To account for this correlation we add the covariance between γ_{USF} and γ_{surf} in error propagation:

$$(\delta D)^2 = \left(\frac{\partial D}{\partial \gamma_{\text{USF}}} \right)^2 (\delta\gamma_{\text{USF}})^2 + \left(\frac{\partial D}{\partial \gamma_{\text{surf}}} \right)^2 (\delta\gamma_{\text{surf}})^2$$
$$+ 2 \frac{\partial D}{\partial \gamma_{\text{USF}}} \frac{\partial D}{\partial \gamma_{\text{surf}}} \text{cov}(\gamma_{\text{USF}}, \gamma_{\text{surf}})$$

Incorporating the covariance, we find $\delta D = 0.015$. It is important to note that this error represents a conservative upper bound. For instance, it is clear from Supplementary Fig. 8 that the largest contribution to the average error in γ_{surf} comes from the region of very high surface energies. Since alloys in this region are also characterized by large γ_{USF} they are expected to be very brittle and hence far from the Pareto front. Consequently, the overall error is small enough to accurately calculate the D-parameter and its concentration dependency. Moreover, smaller deviations encountered during the Pareto front search are further smoothed out through the appropriate choice of the Kernel for the Gaussian process regression and appropriate sampling multiple configuration.

4 Validation of VBA models

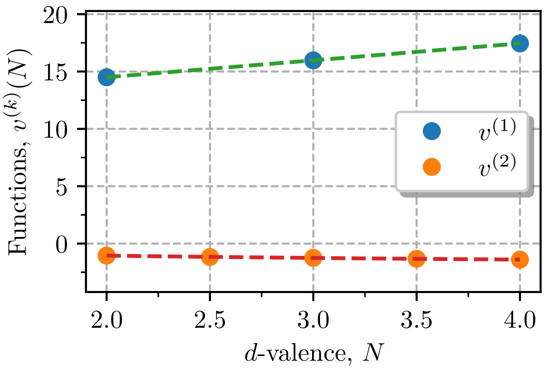

Supplementary Figure 10 Virtual bond functions $v^{(1)}$ and $v^{(2)}$ fitted for the volumes.

4.1 Validation of the virtual bond functions

Supplementary Fig. 10 shows virtual bond functions, $v^{(1)}$ and $v^{(2)}$, fitted for the volumes. They are smooth function of the valences and are fitted with second order polynomials. The misfit volume is directly derived from the volume expansion as follows:

$$\frac{\partial V}{\partial c_i} = v^{(1)}(N_i)w_i + \sum_j 2c_i w_{ij} v^{(2)}(N_{ij}) \quad (1)$$

$$\Delta V_i = \frac{\partial V}{\partial c_i} + \sum_j c_j \frac{\partial V}{\partial c_j} \quad (2)$$

The volume expansion is primarily influenced by the first-order parameter function, $v^{(1)}$. The second-order parameter function $v^{(2)}$ can lead to non-trivial contributions to the equilibrium volume and its concentration derivatives in multi-component alloys. These functions' absolute values are determined by the scaling of the row-dependent prefactors and the band-width factor itself. The row-dependent prefactor for the band-width parameters is optimized for the training data of each individual property. The virtual bond function are not necessarily linear for other properties.

4.2 Material parameter from VBA

Supplementary Fig. 11 displays a comparison between direct and VBA calculations of misfit volumes, where the concentration of Mo in MoNbTi is varied away from the equimolar value. The figure illustrates that the VBA model is capable of capturing the trends in the concentration dependence very well, in contrast to the simple rule-of-mixture that shows significant deviations, especially for decreasing fraction of Mo. This is especially true for alloy systems such as MoNbTi, where relative errors between the VBA model and the rule-of-mixture model are noticeable already for the equimolar system.

However, in the case of the elastic constant, specifically C_{44}, noticeable deviations become apparent when employing the rule-of-mixture approach (as illustrated in Fig. 5a). While the rule-of-mixture does manage to capture the general trend, the discrepancy increase significantly, particularly for the 4-component systems and MoTaW, where it can reach 40 GPa. In contrast, the VBA model showcases good agreement with direct calculations for the majority of the alloys, exhibiting only minor discrepancies for NbTiZr and NbTaTi.

So far we have showed results for quantities that can be directly obtained from CPA calculations. However, we cannot apply the same approach to the surface and USF energies because of the appreciable effect of atomic relaxations. To avoid the need of heavy SQS calculations or of training MTPs for several test alloys we take a simpler route. First, we calculate *unrelaxed* surface and USF energies with CPA for elemental compounds and binary alloys and parameterize the VBA model, as described above. We have checked that the VBA model predicts the CPA values very well. To incorporate relaxation effects, we simply adjust the virtual bond functions to align with *relaxed* surface and USF energies of individual elemental compounds. More precisely, we scale the virtual bond functions by a factor that follows a linear relationship with the d-valences. Such an approach works because fitting to CPA values already includes most of the chemical interactions and the strongest relaxation effects have geometrical nature, which can be taken into account by the rescaling procedure of the universal virtual bond functions.

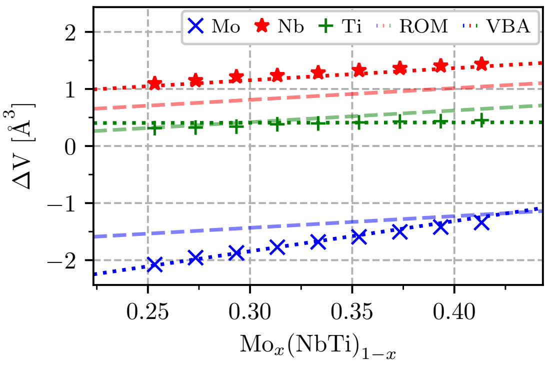

Supplementary Figure 11 Comparison of misfit volumes in MoNbTi with varying concentrations of Mo obtaind from direct calculation ($\star, \times, +$), rule-of-mixture (ROM) and virtual bond approximation (VBA).

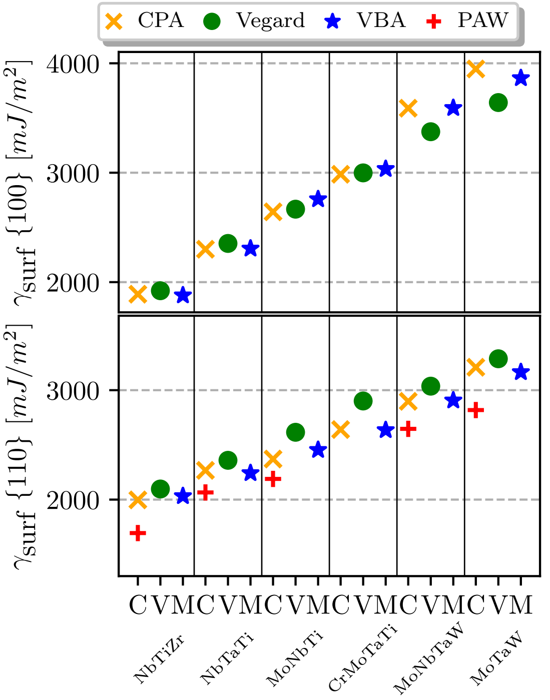

Supplementary Figure 12 Comparison of surface energies obtained from Vegard's law (V), from virtual bond approximation (M) and calculated directly with CPA and PAW (C). PAW is relaxed. CPA unrelaxed. Virtual bond function are not rescaled.

In Supplementary Fig. 12, we compare directly computed surface energies from CPA (without relaxation) and PAW (with relaxations) to our

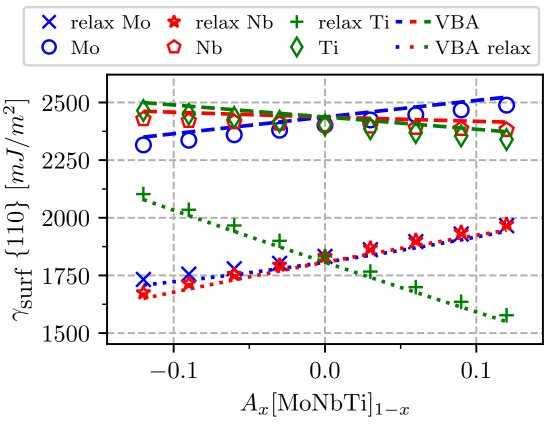

Supplementary Figure 13 Comparison of directly calculated and VBA-model surface energy (110) with and without local relaxations upon concentration changes in equimolar MoNbTi alloy, as A_x[MoNbTi]$_{1-x}$, with x as the molar fraction of A.

model's estimates using ROM and virtual bond approximations. The energies of surfaces and the unstable stacking fault are effectively characterized by VBA model, which outperforms the simpler ROM. Notably, the VBA model significantly enhances the precision of surface energy predictions, underscoring its efficacy in capturing intricate energy behaviors.

The corresponding results for the {110} surface energy varying with the concentration of individual components are shown in Supplementary Fig. 13, from which one can see that the simple rescaling is sufficient to take into account the concentration dependence of relaxation effects.

In Supplementary Fig. 14, we extend our comparative analysis to the elastic constants, following a similar approach. Notably, when examining the elastic constant C_{44}, it becomes evident that the VBA demonstrates a substantial enhancement compared to the traditional ROM. This improvement underscores the efficacy and accuracy of VBA in capturing essential material properties beyond what can be achieved through conventional methods.

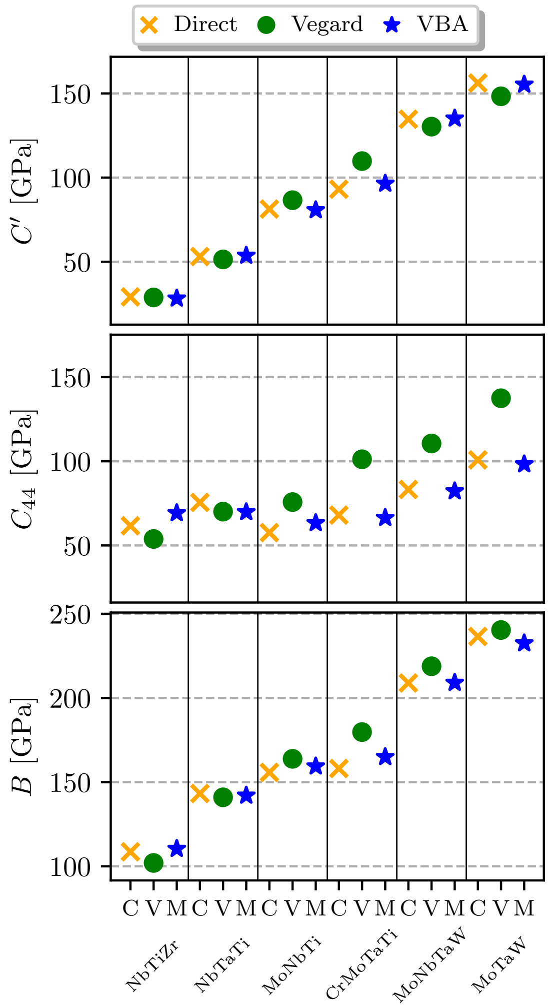

Supplementary Figure 14 Comparison of linear elastic parameters (B, C', C_{44}) obtained from Vegard's law (V), from virtual bond approximation (M) and calculated directly (C).

5 Tabulated Data

Here, we provide data for a broad range of alloys combining values calculated in this work with literature data from Refs. [31–35].

Supplementary Table 1 Comparison of the fracture strain and D parameter calculated from the VBA model. Fracture strain values are from Ref. [31].

Composition	D	Fracture strain [%]
$TiZrV_{0.3}Nb$	1.15	45.00
$TiZrV_{0.3}NbMo_{0.1}$	1.17	45.00
$TiZrV_{0.3}NbMo$	1.28	43.00
$TiZrVNbMo_{0.3}$	1.26	42.00
ZrHfNbTa	1.30	34.00
TiZrNbMo	1.28	33.00
$TiZrVNbMo_{0.5}$	1.29	32.00
$TiZrVNbMo_{0.7}$	1.30	32.00
TiZrVNbMo	1.32	32.00
$TiZrV_{0.25}NbMo$	1.30	30.00
$TiZrVNbMo_{1.3}$	1.34	30.00
TiVNbTaMo	1.48	30.00
TiZrHfVNb	1.19	29.60
$TiZrV_{0.75}NbMo$	1.32	29.00
$Ti_{1.5}ZrHfNbMo$	1.20	28.98
$TiZrV_{0.5}NbMo$	1.31	28.00
$TiZrV_{0.3}NbMo_{0.7}$	1.27	26.60
TiZrVNbMo	1.33	26.00
TiVNbMo	1.39	25.62
$TiZrV_{0.3}NbMo$	1.30	25.00
$TiZrHf_{0.5}NbMo_{0.5}$	1.20	24.61
$TiZrV_3NbMo$	1.37	24.00
$TiZrHfNb_{1.5}Mo$	1.28	23.97
$TiZrV_2NbMo$	1.35	23.00
$TiZrV_{1.5}NbMo$	1.35	20.00
TiVNbTaW	1.49	20.00
$TiZrV_{0.3}NbMo_{1.3}$	1.33	20.00
$TiZr_{0.5}HfNbMo$	1.30	18.02
$TiZrHf_{1.5}NbMo$	1.23	16.83
$TiZr_{1.5}HfNbMo$	1.22	16.09
TiNbTaMoW	1.51	14.10
$TiZrHfNb_{0.5}Mo$	1.22	13.02
$TiZrHf_{0.5}NbMo$	1.29	12.09
$Ti_{0.5}ZrHfNbMo$	1.30	12.08
TiZrHfNbTaMo	1.37	12.00
NbTaVW	1.53	12.00
TiNbTaMoW	1.51	11.50
$TiZrHfNbMo_{1.5}$	1.32	10.83
TiVNbTaMoW	1.51	10.60
TiZrHfNbMo	1.26	10.20
TiZrHfNbMo	1.26	10.12
VNbTaMoW	1.53	8.80
$Ti_{0.75}NbTaMoW$	1.52	8.40
$TiZrV_{0.3}NbMo_{1.5}$	1.36	8.00
$Ti_{0.5}NbTaMoW$	1.53	5.90
NbTaMoW	1.53	2.60
$Ti_{0.25}NbTaMoW$	1.53	2.50
NbTaMoW	1.53	2.10
NbTaMoW	1.53	1.90
VNbTaMoW	1.54	1.70
VNbTaMoW	1.54	1.70

Supplementary Table 2 Comparison of τ_y calculated with the VBA model to experimental ones. Experimental values are from Refs. [32–35].

Composition	τ_{y} Expt.	τ_{y} Pred.
TiZrNbV	322.28	227.65
$TiZrNbVMo_{0.3}$	382.15	284.91
$TiZrNbVMo_{0.5}$	441.70	312.91
$TiZrNbVMo_{0.7}$	517.10	334.93
TiZrNbVMo	521.31	359.43
$TiZrNbVMo_{1.3}$	449.14	376.28
$TiZrNbVMo_{1.5}$	483.77	384.29
$TiZrNbVMo_{1.7}$	497.36	390.25
$TiZrNbVMo_{2.0}$	536.20	396.08
$TiZrNbV_{0.3}$	245.26	202.24
$TiZrNbV_{0.3}Mo_{0.1}$	266.62	236.27
$TiZrNbV_{0.3}Mo_{0.3}$	389.60	291.20
$TiZrNbV_{0.3}Mo_{0.5}$	386.04	332.78
$TiZrNbV_{0.3}Mo_{0.7}$	429.72	364.46
$TiZrNbV_{0.3}Mo$	435.87	398.41
$TiZrNbV_{0.3}Mo_{1.3}$	483.77	420.65
$TiZrNbV_{0.3}Mo_{1.5}$	475.03	430.72
NbTiZr	280.53	177.43
NbTiZrV	324.22	227.65
NbTiZrVMo	453.67	359.43
TiZrHfNbV	401.89	185.11
TiZrHfNbCr	545.91	485.38
NbTaTi	190.00	32.75
MoNbTi	300.00	206.73
CrMoTaTi	605.00	443.13
MoNbTaW	300.00	226.90

References

[1] Zheng, H. *et al.* Multi-scale investigation of short-range order and dislocation glide in MoNbTi and TaNbTi multi-principal element alloys. *npj Comput. Mater.* **9**, 89 (2023).

[2] Singh, P. *et al.* Design of high-strength refractory complex solid-solution alloys. *npj Comput. Mater.* **4**, 16 (2018).

[3] Inoue, A., Zhang, T. & Masumoto, T. Reductilization of embrittled la-al-ni amorphous alloys by viscous flow deformation in a supercooled liquid region. *J. Non-Cryst. Solids* **156-158**, 598–602 (1993).

[4] Kalali, D. G. *et al.* Role of ti on the microstructure and mechanical properties of monbti medium-entropy alloy. *Int. J. Refract. Met. Hard Mater* **118**, 106487 (2024).

[5] Startt, J., Kustas, A., Pegues, J., Yang, P. & Dingreville, R. Compositional effects on the mechanical and thermal properties of monbtati refractory complex concentrated alloys. *Mater. Design* **213**, 110311 (2022).

[6] Zhang, E. *et al.* On phase stability of mo-nb-ta-w refractory high entropy alloys. *Int. J. Refract. Met. Hard Mater* **103**, 105780 (2022).

[7] Mak, E., Yin, B. & Curtin, W. A ductility criterion for bcc high entropy alloys. *J. Mech. Phys. Solids* **152**, 104389 (2021).

[8] Tseng, K.-K. *et al.* Effects of mo, nb, ta, ti, and zr on mechanical properties of equiatomic hf-mo-nb-ta-ti-zr alloys. *Entropy-switz.* **21** (2019).

[9] Zhang, Y., Wei, Q., Xie, P. & Xu, X. An ultrastrong niobium alloy enabled by refractory carbide and eutectic structure. *Mater. Res. Lett.* **11**, 169–178 (2023).

[10] Dobbelstein, H., Gurevich, E. L., George, E. P., Ostendorf, A. & Laplanche, G. Laser metal deposition of compositionally graded tizrnbta refractory high-entropy alloys using elemental powder blends. *Addit. Manuf.* **25**, 252–262 (2019).

[11] Maresca, F. & Curtin, W. A. Mechanistic origin of high strength in refractory bcc high entropy alloys up to 1900K. *Acta Mater.* **182**, 235–249 (2020).

[12] Holec, D. *et al.* Macroscopic elastic properties of textured zrn-aln polycrystalline aggregates: From ab initio calculations to grain-scale interactions. *Phys. Rev. B* **90**, 184106 (2014).

[13] Song, H. *et al.* Local lattice distortion in high-entropy alloys. *Phys. Rev. Mater.* **1**, 023404 (2017).

[14] Tian, L.-Y. *et al.* Elastic constants of random solid solutions by sqs and cpa approaches: the case of fcc ti-al. *J. Phys. Condens. Matter* **27**, 315702 (2015).

[15] Moitzi, F., Romaner, L., Ruban, A. V. & Peil, O. E. Accurate ab initio modeling of solid solution strengthening in high entropy alloys. *Phys. Rev. Mater.* **6**, 103602 (2022).

[16] Elder, K. L. M. *et al.* Computational discovery of ultra-strong, stable, and lightweight refractory multi-principal element alloys. part i: design principles and rapid down-selection. *npj Comput. Mater.* **9**, 84 (2023).

[17] Khatamsaz, D. *et al.* Multi-objective materials bayesian optimization with active learning of design constraints: Design of ductile refractory multi-principal-element alloys. *Acta Mater.* **236**, 118133 (2022).

[18] Rao, S. *et al.* Solution hardening in body-centered cubic quaternary alloys interpreted using suzuki's kink-solute interaction model. *Scripta Mater.* **165**, 103–106 (2019).

[19] Rao, S., Woodward, C., Akdim, B., Senkov, O. & Miracle, D. Theory of solid solution strengthening of bcc chemically complex alloys. *Acta Mater.* **209**, 116758 (2021).

[20] Xu, S., Chavoshi, S. Z. & Su, Y. On calculations of basic structural parameters in

multi-principal element alloys using small atomistic models. *Nato. Sc. S. Ss. Iii. C. S.* **202**, 110942 (2022).

[21] Senkov, O., Gorsse, S. & Miracle, D. High temperature strength of refractory complex concentrated alloys. *Acta Mater.* **175**, 394–405 (2019).

[22] Senkov, O., Miracle, D. & Rao, S. Correlations to improve room temperature ductility of refractory complex concentrated alloys. *Materials Science and Engineering: A* **820**, 141512 (2021).

[23] Coury, F. G., Kaufman, M. & Clarke, A. J. Solid-solution strengthening in refractory high entropy alloys. *Acta Mater.* **175**, 66–81 (2019).

[24] Cordero, Z. C., Knight, B. E. & Schuh, C. A. Six decades of the hall-petch effect - a survey of grain-size strengthening studies on pure metals. *Int. Mater. Rev.* **61**, 495–512 (2016).

[25] Maresca, F. & Curtin, W. A. Theory of screw dislocation strengthening in random bcc alloys from dilute to high-entropy alloys. *Acta Mater.* **182**, 144–162 (2020).

[26] Baruffi, C., Maresca, F. & Curtin, W. A. Screw vs. edge dislocation strengthening in body-centered-cubic high entropy alloys and implications for guided alloy design. *MRS Commun.* **12**, 1111–1118 (2022).

[27] Melia, M. A. *et al.* High-throughput additive manufacturing and characterization of refractory high entropy alloys. *Appl. Mater. Today* **19**, 100560 (2020).

[28] Andric, P. & Curtin, W. A. Atomistic modeling of fracture. *Model. Simul. Mater. Sc.* **27**, 013001 (2018).

[29] Hodapp, M. & Shapeev, A. Machine-learning potentials enable predictive and tractable high-throughput screening of random alloys. *Phys. Rev. Mater.* **5**, 113802 (2021).

[30] Larsen, P. M., Schmidt, S. & Schiøtz, J. Robust structural identification via polyhedral template matching. *Model. Simul. Mater. Sc.* **24**, 055007 (2016).

[31] Singh, P. *et al.* A ductility metric for refractory-based multi-principal-element alloys. *Acta Mater.* **257**, 119104 (2023).

[32] Wu, Y. *et al.* Enhanced electrochemical performance by wrapping graphene on carbon nanotube/sulfur composites for rechargeable lithium-sulfur batteries. *Mater. Lett.* **137**, 277–280 (2014).

[33] Wu, Y. *et al.* Phase composition and solid solution strengthening effect in tizrnbmov high-entropy alloys. *Mater. Design* **83**, 651–660 (2015).

[34] Fazakas, A. *et al.* Experimental and theoretical study of Ti20Zr20Hf20Nb20X20 (x=v or cr) refractory high-entropy alloys. *Int. J. Refract. Met. Hard Mater* **47**, 131–138 (2014).

[35] Xiong, W., Guo, A. X., Zhan, S., Liu, C.-T. & Cao, S. C. Refractory high-entropy alloys: A focused review of preparation methods and properties. *J. Mater. Sci. Technol.* **142**, 196–215 (2023).
